# Cooperation, *cis*-interactions, versatility and evolutionary plasticity of multiple *cis*-acting elements underlie *krox20* hindbrain regulation

**DOI:** 10.1371/journal.pgen.1007581

**Published:** 2018-08-06

**Authors:** Patrick Torbey, Elodie Thierion, Samuel Collombet, Anne de Cian, Carole Desmarquet-Trin-Dinh, Mathilde Dura, Jean-Paul Concordet, Patrick Charnay, Pascale Gilardi-Hebenstreit

**Affiliations:** 1 Institut de Biologie de l’Ecole normale supérieure (IBENS), Ecole normale supérieure, CNRS, Inserm, PSL Université, Paris, France; 2 Sorbonne Universités, Muséum National d’Histoire Naturelle, CNRS UMR 7196, INSERM U1154, Paris, France; Stowers Institute for Medical Research, UNITED STATES

## Abstract

*Cis*-regulation plays an essential role in the control of gene expression, and is particularly complex and poorly understood for developmental genes, which are subject to multiple levels of modulation. In this study, we performed a global analysis of the *cis*-acting elements involved in the control of the zebrafish developmental gene *krox20*. *krox20* encodes a transcription factor required for hindbrain segmentation and patterning, a morphogenetic process highly conserved during vertebrate evolution. Chromatin accessibility analysis reveals a *cis*-regulatory landscape that includes 6 elements participating in the control of initiation and autoregulatory aspects of *krox20* hindbrain expression. Combining transgenic reporter analyses and CRISPR/Cas9-mediated mutagenesis, we assign precise functions to each of these 6 elements and provide a comprehensive view of *krox20 cis*-regulation. Three important features emerged. First, cooperation between multiple *cis*-elements plays a major role in the regulation. Cooperation can surprisingly combine synergy and redundancy, and is not restricted to transcriptional enhancer activity (for example, 4 distinct elements cooperate through different modes to maintain autoregulation). Second, several elements are unexpectedly versatile, which allows them to be involved in different aspects of control of gene expression. Third, comparative analysis of the elements and their activities in several vertebrate species reveals that this versatility is underlain by major plasticity across evolution, despite the high conservation of the gene expression pattern. These characteristics are likely to be of broad significance for developmental genes.

## Author summary

Animal development relies on the early delimitation of specific embryonic territories that will later participate in the formation of tissues and organs. This process is governed by sets of so-called developmental genes. The activities of the genes are themselves controlled by associated DNA sequences called enhancers. In vertebrates a part of the embryonic brain is delimited by the activity of the gene *krox20*. In this study, we have performed a comprehensive analysis of the *krox20* regulatory landscape in the zebrafish vertebrate model. We show that 6 enhancers cooperate according to different modes to establish the complete pattern of *krox20* activity. Furthermore, these enhancers appear unexpectedly versatile, combining different types of activities. This versatility is underlain by major plasticity across vertebrate evolution, despite the high conservation of the delimitation process. These observations are likely to be of broad significance for developmental genes.

## Introduction

Enhancers are short, *cis-*acting regulatory elements that modulate transcription of target genes, relatively independently of their orientation or distance with respect to the promoter. They act as platforms to recruit multiple transcription factors [1] that interact with the transcription machinery at the promoter via cofactors [2]. A single gene can be controlled by multiple enhancers that show different activity profiles, providing both diversity and specificity of expression [3], or redundant profiles that may be required to ensure transcriptional robustness [4]. Interactions between enhancers can occur through different modes of cooperation: additive, synergistic, repressive, hierarchical or competitive [5]. Multiplicity of enhancers is a common feature among developmental genes [6] and is likely to play a major role in the evolution of gene expression, as it provides the necessary flexibility for pattern evolution [7].

For many years, the functions of enhancers have been mainly investigated through analysis of transgenic constructs carrying a reporter gene driven by a minimal promoter and linked to the enhancer [8]. Although fruitful, this approach is based upon the assumption that enhancer function can be recapitulated by the activity profile deduced from such an assay. However, it has not been established that this is always the case. In recent years, the advent of easy and efficient genome editing techniques, in particular those based on the CRISPR/Cas9 system, have facilitated mutation of putative enhancers in their natural genomic context [9,10], enabling the direct dissection of enhancer function in various species, including vertebrates [11].

Hindbrain segmentation is a highly conserved morphogenetic process in vertebrate development [12]. Among the regulatory genes involved in segmentation, *Krox20* (also known as *Egr2*) plays a particularly important role. It encodes a zinc-finger transcription factor and is specifically and precisely expressed in two developing hindbrain segments, rhombomeres (r) 3 and 5 [13–15]. Krox20 is responsible for the formation and specification of these rhombomeres [16–19]. The regulation of *Krox20* expression in the developing hindbrain provides an attractive model to study the functions and evolution of *cis*-acting elements involved in control of patterning in vertebrates. Three evolutionarily conserved enhancer elements active in the hindbrain have previously been identified near the *Krox20* gene, termed A, B and C [20]. Analysis of chicken element A revealed that it is active in r3 and r5 and requires Krox20 binding for this activity [20], suggesting that it acts as an autoregulatory element. Indeed, deletion of element A in the mouse leads to a complete loss of *Krox20* expression at late stages without affecting early stages, a phenotype very similar to *Krox20* loss-of-function [21]. In contrast, chicken element B enhancer activity is Krox20-independent, and is restricted to r5 [20,22], making it a prime candidate for the initiation of *Krox20* expression in r5. Finally, chicken enhancer C is active in the r3-r5 region, also in a Krox20-independent manner, suggesting that it might contribute to the initiation of *Krox20* expression in r3 and/or r5 [20,23]. Surprisingly, deletion of element C in the mouse does not affect *Krox20* expression at early stages, but leads to a loss of maintenance of *Krox20* at late stages in r3 [24]. This loss of maintenance is due to cooperation in *cis* between element A and C, leading to increased accessibility of element A and potentiation of its autoregulatory activity in r3 [24]. This unexpected function of element C, unlike a classical enhancer, clearly illustrates the necessity of mutating putative *cis*-regulatory elements in their chromosomal context to decipher their true function.

In spite of these observations, previous analyses do not provide a complete global picture of *Krox20* regulation in the hindbrain. In particular, they do not explain the basis for early *Krox20* expression in r3. We therefore decided to engage in a systematic search and analysis of *Krox20 cis*-regulatory elements. For this purpose, we turned to the zebrafish, which allows easier identification and functional characterisation of regulatory elements and evolutionary comparisons with existing data from other vertebrates.

This approach has revealed a complex *cis*-regulatory landscape, with 6 elements controlling zebrafish *krox20* expression in the hindbrain. Three of these are the homologues of the previously identified mouse and chicken elements A, B and C. Combining transgenic reporter analyses and CRISPR/Cas9-mediated mutagenesis in the chromosomal context, we assign precise functions to each of these 6 elements and provide a comprehensive view of *krox20 cis*-regulation in the hindbrain. Three important features of gene regulation emerge. First, cooperation and redundancy between multiple *cis*-elements play a major role in regulation (for instance, 4 elements cooperate to maintain autoregulation). Second, unexpected versatility of several elements allows them to be involved in different aspects of expression control. Third, this versatility is underlain by major plasticity across vertebrate evolution, despite the highly conserved pattern of *Krox20* expression. These characteristics of *Krox20 cis*-regulation are likely shared by other developmental genes and are therefore of broad significance.

## Results

### Zebrafish hindbrain transcriptional enhancers in the *krox20* locus identified by chromatin accessibility analysis

**Fig 1:**
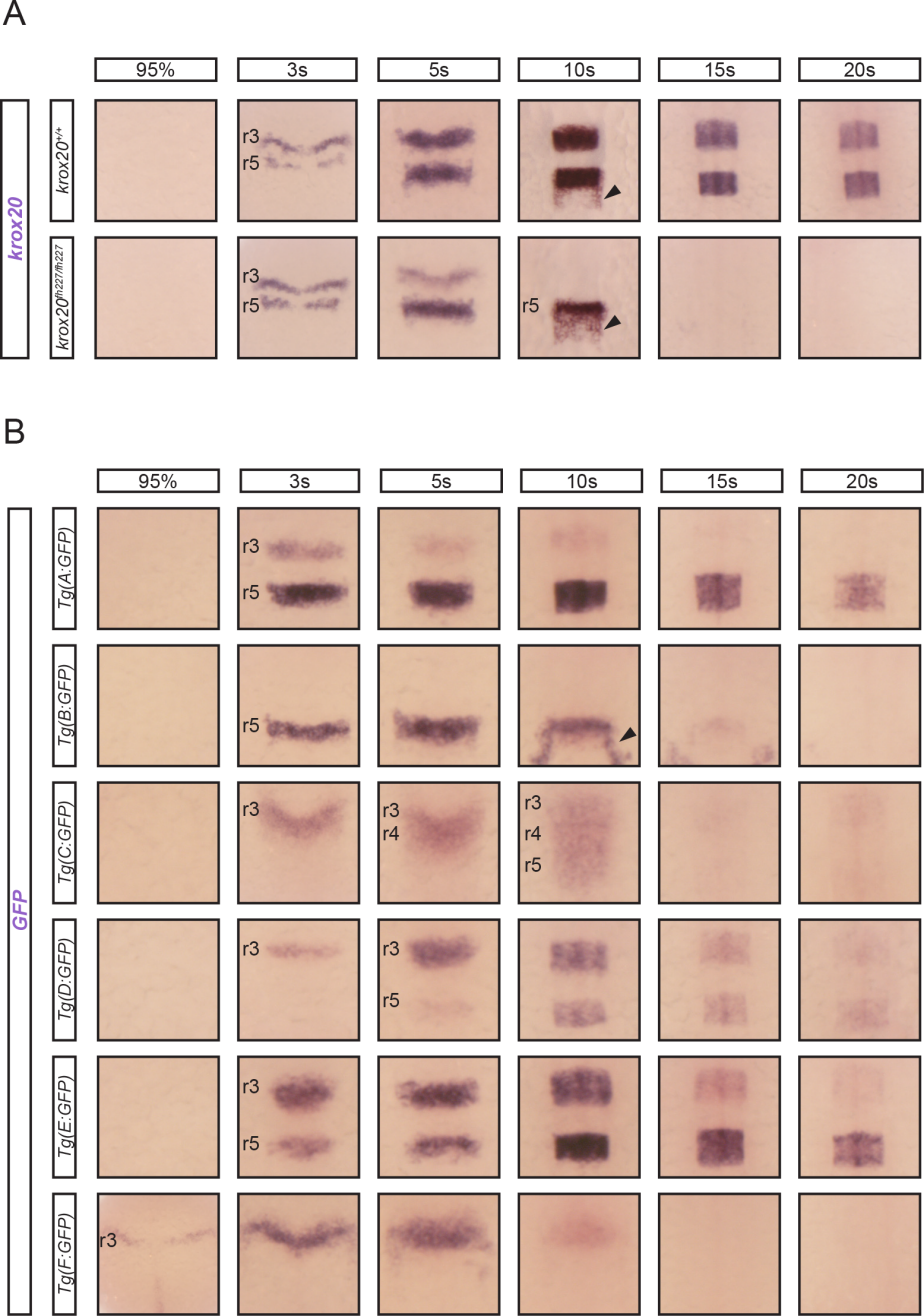
*krox20* expression and enhancer dynamics. (A) Analysis of *krox20* expression by in situ hybridization at the indicated somite stages (s) in wild type (*krox20*^*+/+*^) or *krox20* null (*krox20*^*fh227/fh227*^) backgrounds. (B) Analysis of *GFP* expression by in situ hybridization at the indicated stages in 6 transgenic lines carrying GFP reporter constructs in which the different putative *krox20* enhancers have been inserted. Positions of r3, r4 and r5 are shown. Neural crest cells migrating from r5/r6 are indicated by arrowheads.

To study *krox20 cis*-regulation in detail in the zebrafish hindbrain, we first analysed its expression pattern by *in situ* hybridization, to provide a reference for comparison with the activities of putative enhancers. As *krox20* regulation in the hindbrain has been shown to involve a positive feedback loop [20], we examined both wild type embryos and those carrying a homozygous point mutation in the *krox20* coding sequence that abolishes Krox20 function and thereby prevents autoregulation (*krox20*^*fh227*^ allele [21,25]). In agreement with previous studies [21], *krox20* expression is dynamic between the 95% epiboly and 20-somite stages (20s). A positive feedback loop contributes to the amplification and maintenance of expression, as in absence of active protein, *krox20* mRNA disappears from r3 between 5s and 10s, and from r5 between 10s and 15s (Fig 1A). In contrast, in the wild type, the mRNA is maintained in both rhombomeres beyond 20s. *krox20* is also expressed in neural crest cells leaving the neural tube from the r5/r6 region (Fig 1A, arrowhead).

**Fig 2:**
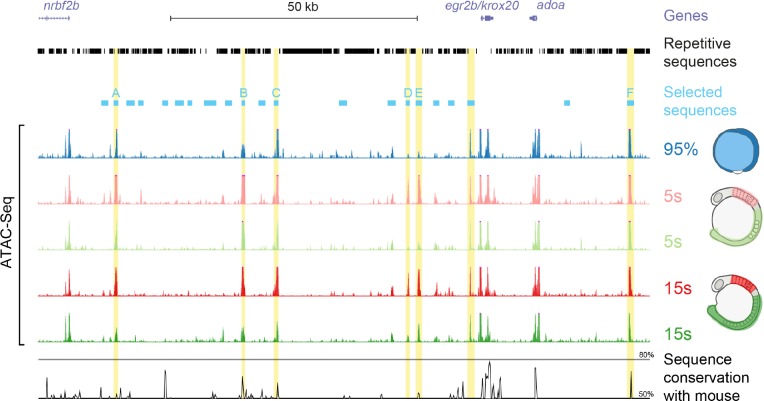
DNA accessibility and candidate enhancer sequences within and around the zebrafish *krox20* locus. UCSC genome browser view of the *krox20* locus showing gene positions (purple), repetitive sequences (black) and the sequences selected for enhancer activity tests (light blue), including those that showed activity (named A to F). Below are ATAC-seq data from experiments performed at the indicated stages, either on whole embryos (95% epiboly) or dissected hindbrain or posterior regions of the embryos (5s and 15s), as shown on the schematics on the right side. The seven mostly significant peaks located in non-coding sequences are highlighted in yellow. Underneath is a Vista browser view of sequence conservation between zebrafish and mouse (black) over the region.

To identify the transcriptional enhancers responsible for *krox20* expression in the hindbrain, we undertook a systematic approach based on the observation that active *cis*-regulatory sequences typically show greater DNA accessibility than other sequences. We assessed chromatin accessibility within the *krox20* locus and its vicinity by ATAC-seq [26]. ATAC-seq was performed on either 95% epiboly whole embryos or on micro-dissected regions (whole hindbrain, including r3 and r5, or a posterior region devoid of *krox20*-expressing cells; Fig 2) from 5s and 15s embryos. These conditions correspond to key moments in *krox20*’s expression dynamics: at the very beginning of gene activation (95% epiboly), after activation with limited (5s hindbrain) or full (15s hindbrain) contributions of the autoregulatory loop, and in regions where the gene remains silent (posterior regions). The analysis revealed 7 major peaks that are present outside of the promoter and coding sequence when *krox20* is active (Fig 2). As all 7 peaks were located in non-repetitive regions and additional enhancers may have been missed by ATAC-Seq, we extended our survey of functional enhancers to all non-repetitive intergenic regions. This led to the selection of 22 sequences (ranging from 720 to 1726 bp), 7 containing one of the identified accessibility peaks (Fig 2, blue boxes).

To evaluate transcriptional enhancer activities associated with the 22 selected sequences, each was cloned into the Zebrafish Enhancer Detection (ZED) plasmid [27], upstream of a GFP reporter gene driven by the *gata2* minimal promoter. These constructs were co-injected with transposase mRNA into one-cell stage zebrafish embryos and GFP fluorescence was monitored. Among the 22 cloned sequences, 6 led to hindbrain-specific *GFP* expression (Fig 1B), suggesting that each harboured a transcriptional enhancer. These 6 sequences were named A to F according to their positions along the locus. Each sequence included one of the accessibility peaks, demonstrating that assessment of chromatin accessibility by ATAC-seq is a powerful approach to identify *cis*-regulatory elements (Fig 2). All of these peaks (with the exception of the one corresponding to element F) were reduced at 15s in the krox20-negative posterior region of the embryo and two of them (corresponding to elements D and E) were very small at 95% epiboly (Fig 2), suggesting that in most of these regions, DNA accessibility is correlated with gene activity. In silico analysis of the 7th accessible region, located close to the promoter, revealed a putative binding site for the architectural protein CTCF [28] that may participate in increasing chromatin accessibility.

Elements A to E are located upstream of *krox20*, whereas element F is located downstream. Elements A, B, C and F show sequence similarity with the previously identified mouse and chicken hindbrain enhancers A, B and C [20] and the mouse NE element [24], respectively, and occupy the same relative positions along the locus (Fig 2). Sequence conservation between species is relatively high for elements B and F, reduced for element C, and low for element A (Figs 2 and S1). Sequences weakly homologous to element E were also identified in the vicinity of the mouse and chicken *krox20* gene, again at the same relative positions (Figs 2 and S1). No sequences homologous to element D were detected in the mouse or chick (Fig 2).

To further investigate the activity of the 6 zebrafish elements, embryos injected with each construct were used to generate stable transgenic lines, whose profiles of *GFP* expression during hindbrain development were established by in situ hybridization (Fig 1B). At least two independent lines were analysed for each element, with the exception of element F, for which only one line was obtained. The patterns of *GFP* expression were identical for the different lines corresponding to the same element. We found that element A is weakly active in r3 between 3s and 10s, and much stronger in r5 from 3s to beyond 20s. Element B is active only in r5 between 3s and approximately 10s. At 10s, element B also drives *GFP* expression in neural crest cells migrating posteriorly to r5 (Fig 1B). Element C activity, first observed in r3 at 3s, later extends into r4 at 5s and then into r5 at 10s, and vanishes thereafter. Elements D and E are both active in r3 and r5 between 3s and 20s; D is more efficient in r3 at early stages, whereas E shows more activity in r5 at late stages. Finally, element F activity is restricted to r3, with very early onset (95% epiboly) but rapid extinction (at around 10s).

This enhancer assay suggests that among the 22 non-repetitive intergenic sequences located within and around the *krox20* locus, 6 are likely to have hindbrain enhancer activities that reflect aspects of the normal hindbrain expression of the gene. This conclusion is further supported in that 3 of these elements, A, B and C, appear to show both structural and functional homology to previously characterised mouse and chicken enhancers [20]. Indeed, the patterns of activity of the homologous elements in the three species are very similar: B is restricted to r5, A is active in both r3 and r5, and C is active in a domain extending from r3 to r5. Sequences homologous to elements E and F also occur in the chick and mouse genomes, at the same relative positions as in the zebrafish. Together, the 6 zebrafish *cis*-acting elements appear to recapitulate all aspects of *krox20* expression, in particular early activity in r3 and r5 for F and B, respectively, and intermediate or late activities in both r3 and r5 for all others. Finally, the fact that almost all major accessibility peaks identified by ATAC-seq correspond to hindbrain enhancers constitutes a strong validation of the use of this procedure to identify novel transcriptional *cis*-acting elements.

### Deletion of endogenous enhancers reveals those required for the initiation of *krox20* expression

To determine the roles played in *krox20* hindbrain regulation by the various *cis*-acting elements identified in the vicinity of the gene, we generated stable zebrafish lines with deletions of each element using CRISPR/Cas9 technology. Mutations were obtained by injecting into one-cell stage embryos the Cas9 protein together with two guide RNAs that targeted sequences flanking each element, resulting in its deletion. Stable lines were then selected; the deletions were characterised by PCR cloning and sequencing (S2 Fig) and the lines were used to obtain homozygous mutant embryos. The generation of stable lines carrying deletions of several elements was sometimes problematic. In such cases, we used an alternative approach that allowed us to obtain mutations in both alleles, directly in the injected embryo. Embryos were injected with the Cas9 protein, together with a mix of 3–4 guide RNAs that targeted evolutionarily conserved short sequences and/or putative binding sites for transcription factors, located within a 150–450 bp region presumably corresponding to the core enhancer (S2 Fig). This procedure was very efficient, allowing the introduction of deletions within both alleles at the same time, as demonstrated by the absence of fragments corresponding to the wild type allele following PCR amplification and further analysis of the DNA sequences (S3 Fig). Although the deletions introduced in both alleles might be different (S3B Fig), in all the cases analysed they led to complete or almost complete inactivation of the element, as judged by the homogeneity of the phenotypes associated with the mutations and their similarity to those corresponding to germ-line deletion of the same element (S4 Fig). Genotypes of mutated embryos generated through this approach (somatic deletion) are noted with the * symbol following the inactivated element.

**Fig 3:**
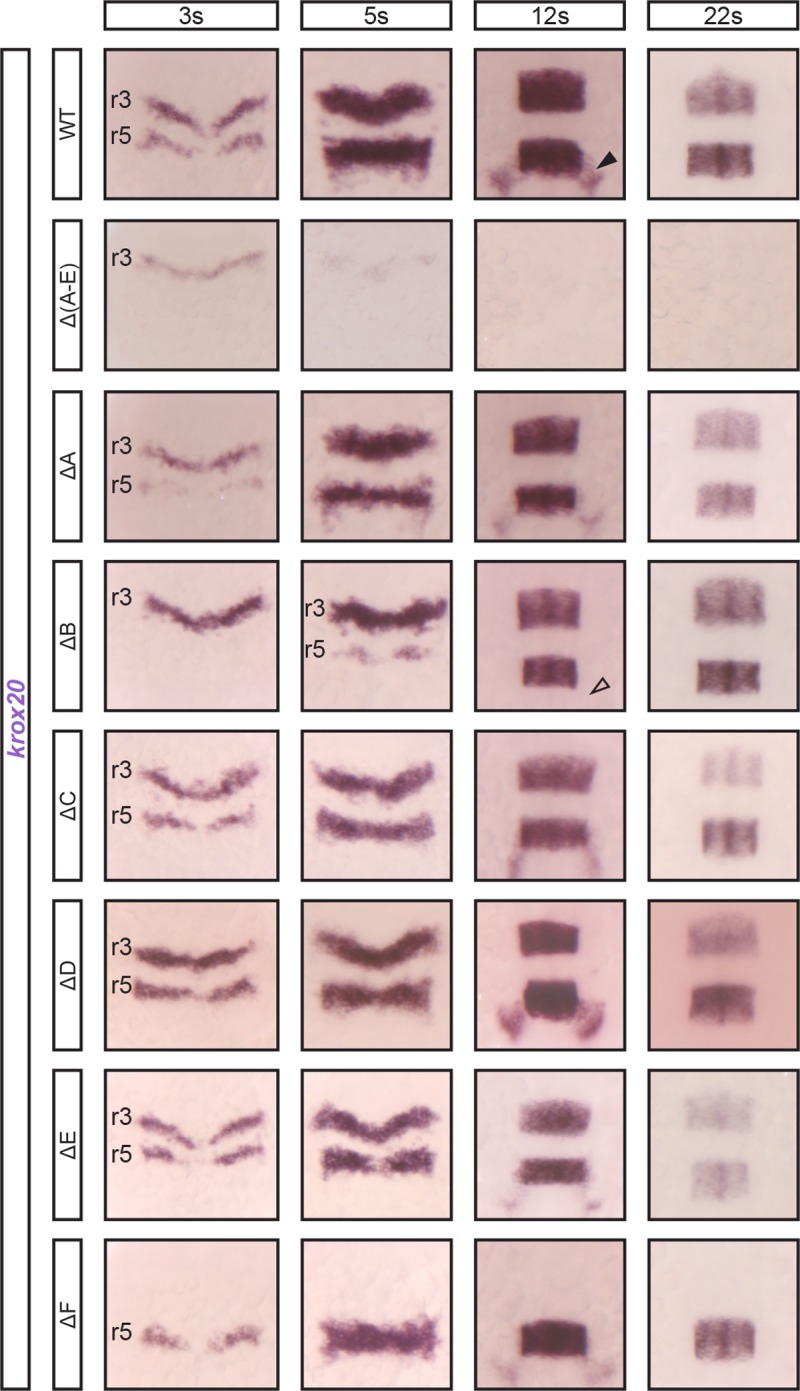
Consequences of single enhancer deletions on *krox20* expression. Embryos from lines carrying homozygous deletions (∆) of a single enhancer element or of a large region, ∆(A-E), were analysed for the expression of *krox20* by in situ hybridization at the indicated stages. WT, wild type. Positions of r3 and r5 are shown. Neural crest cells migrating from r5/r6 are indicated by arrowheads (black: presence, transparent: absence).

To grossly map *cis*-acting elements governing *krox20* in the hindbrain, we first generated a line carrying a deletion, ∆(A-E), that completely eliminated a 75 kb intergenic region between *krox20* and *nrbf2*, including the 5 identified upstream elements, but excluding the *krox20* promoter region (S2 Fig). The expression of *krox20* in embryos carrying a homozygous ∆(A-E) deletion was dramatically affected: *krox20* expression was initiated in r3, but *krox20* mRNA levels rapidly decreased in this rhombomere and no expression was ever observed in r5 (Fig 3, ∆(A-E)). This result indicates that *cis*-acting sequences sufficient for initiation of *krox20* expression in r3 are located outside of the deleted region. In contrast, *cis*-acting elements necessary for initiation in r5 and maintenance in r3 are located within this region.

There is an obvious candidate for governing *krox20* initiation in r3: the downstream element F, which shows enhancer activity at early stages specifically in this rhombomere (Fig 1). Indeed, in homozygous mutants with a deletion of element F, *krox20* expression was completely abolished in r3 at all stages, whereas r5 expression was unaffected (Fig 3, ∆F). Therefore, element F is absolutely required for initiation of *krox20* expression in r3, consistent with its enhancer activity there at early stages (Fig 1B). In absence of any initiation, the feedback loop cannot be engaged and so no expression is observed at later stages either.

We next sought to identify the *cis*-acting sequences involved in the initiation of *krox20* expression in r5 that are located within the ∆(A-E) deleted region. For this purpose, we generated zebrafish lines carrying deletions of each of the elements A, B, C, D or E. No phenotype was observed with any deletion in the heterozygous state. When affecting both alleles, two deletions, ∆A and ∆B, appeared to delay initiation of *krox20* expression in r5 (Fig 3). In the case of ∆B, r5 expression was completely abolished at 3s and dramatically reduced at 5s, but at later stages, normal levels of expression were gradually reached (Fig 3). There was no effect on r3. Note that B is the only element whose deletion also obliterates *krox20* expression in neural crest cells derived from the r5/r6 region (Fig 3, arrowhead). For ∆A, r5 expression was also affected at 3s and 5s, although less severely than in the ∆B mutant. However, ∆A also led to a slight reduction of expression in both r3 and r5 at later stages. The other deletions (∆C, ∆D and ∆E) did not affect *krox20* expression in r5 at early stages (Fig 3).

**Fig 4:**
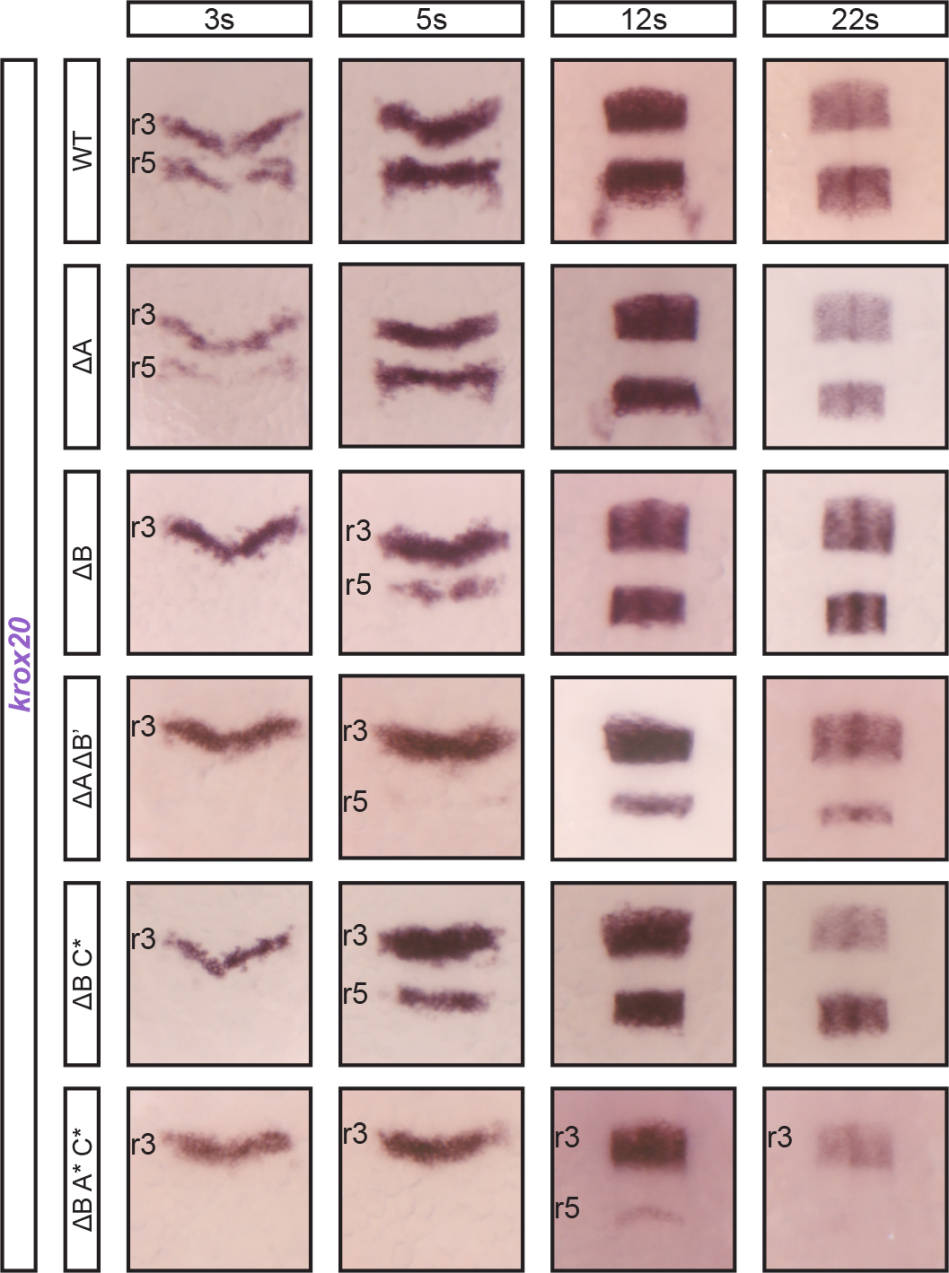
*krox20* r5 expression involves cooperation between three enhancer elements. Embryos carrying combinations of deletions affecting both alleles of elements B, A and/or C, as indicated, were analysed for *krox20* expression by in situ hybridization at the indicated stages. Somatic deletions are indicated by the * symbol and positions of r3 and r5 are shown.

**Fig 5:**
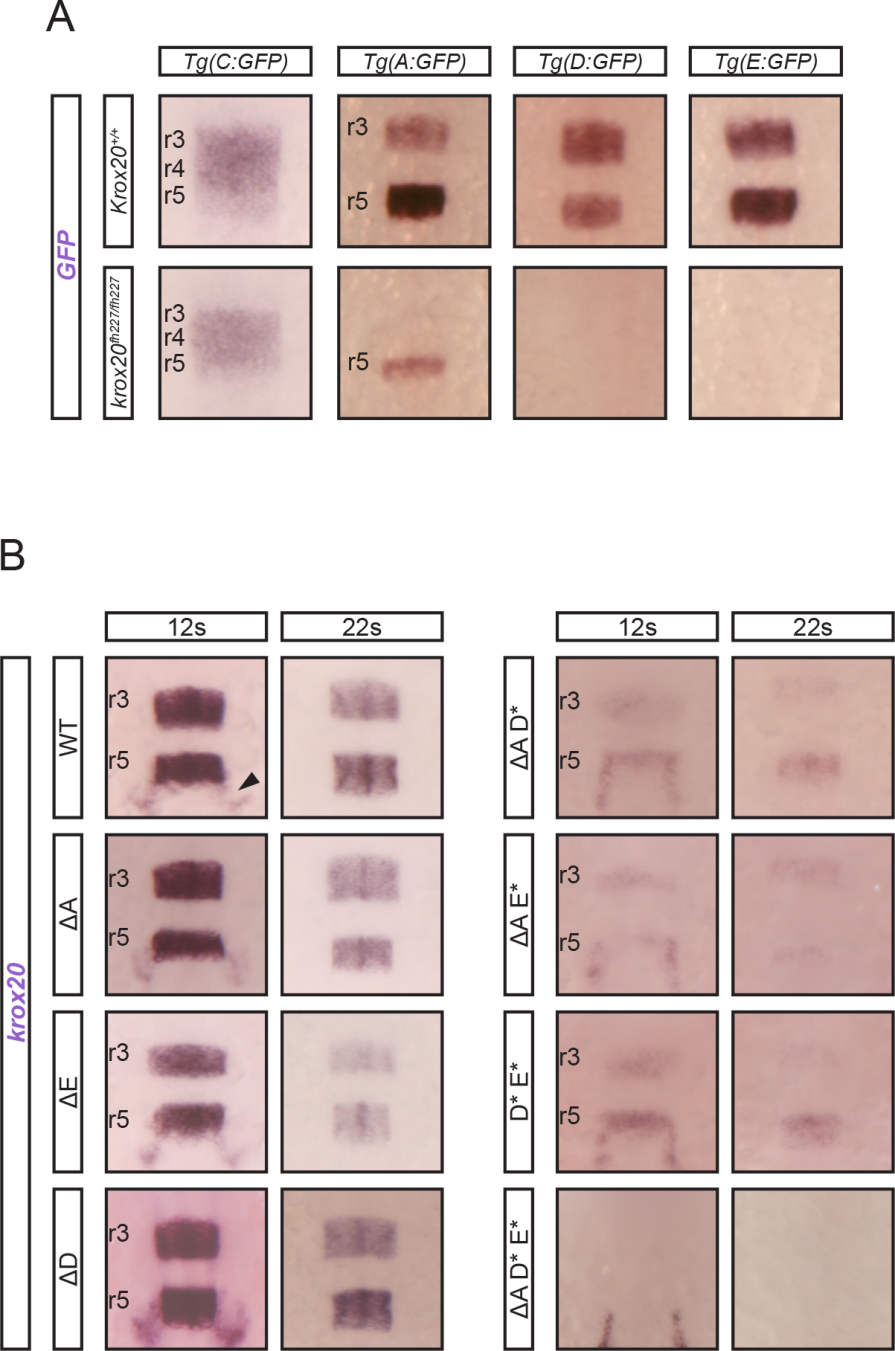
Three enhancer elements cooperate for *krox20* positive autoregulation. (A) Analysis of the dependence on Krox20 of the enhancer elements affecting late *krox20* expression. Four transgenes consisting of GFP reporter constructs, in which the indicated *krox20* enhancers were inserted, were transferred into wild type (*krox20*^*+/+*^) and *krox20* null (*krox20*^*fh227/fh227*^) backgrounds and embryos were analysed for *GFP* expression by in situ hybridization in at the 12s stage. Positions of r3, r4 and r5 are shown. (B) Embryos carrying combinations of deletions affecting both alleles of elements A, D and/or E, as indicated, were analysed for *krox20* expression by in situ hybridization at the indicated stages. Somatic deletions are indicated by the * symbol and positions of r3 and r5 are shown. Neural crest cells migrating from r5/r6 are indicated by an arrowhead.

To determine whether elements A and B are the only contributors to the initiation of *krox20* expression in r5, we examined the effect of deleting both, by introducing a deletion of B (∆B’) in a ∆A background (S2 Fig). Embryos carrying homozygous deletions of both elements (∆A ∆B’) show a stronger phenotype than embryos with a single mutation: expression in r5 is only detected after 5s and late expression is also severely affected, presumably because the feedback loop cannot be appropriately established due to late and very poor initiation (Fig 4). As there was still limited expression maintained in r5 in the double mutant, we wondered whether a third element might be involved in the initiation step. Three elements show enhancer activity in r5: C, D and E (Fig 1). However, for elements D and E, this activity appears to be totally dependent on the presence of functional Krox20 protein (Fig 5A). This is not the case for element C, raising the possibility that it could cooperate with elements A and B to initiate *krox20* in r5. We therefore combined deletions in C with deletions of A and/or B and examined whether any expression remained. The combination of homozygous B and C deletions did not increase the severity of the phenotype associated with B deletions (Fig 4). However, the combination of homozygous A, B and C deletions led to an almost complete loss of expression in r5: only a very low level of mRNA was reproducibly observed at 12s (Fig 4).

In conclusion, this analysis identified the *cis*-acting elements involved in the initiation phase of *krox20* expression [21]: their homozygous mutation affects the hindbrain expression of *krox20* at very early stages (at around 3s), before any significant involvement of the autoregulatory loop (Fig 1A). In r3, a single element, F, is absolutely required. In r5, however, the situation is more complex and elements show partial redundancy. Although element B appears as the major contributor, elements A and C are also involved and the mutation of all three elements is required to essentially abolish *krox20* r5 expression. Residual expression could be due to very weak activity of a non-characterised fourth element or to the fact that the internal mutations in enhancers A and C do not totally inactivate them (Figs 4 and S2). Finally, among the identified elements, in the neural crest derived from r5/r6, enhancer B is the only one required for *krox20* expression.

### Characterisation of the *cis*-acting elements controlling *krox20* autoregulation

Three of the *krox20 cis*-acting elements, A, D and E, appear to share similar characteristics: they act as enhancers in both r3 and r5, and are active at late stages (up to 20s). Furthermore, deletions ∆A and ∆E lead to a slight decrease in *krox20* mRNA levels in both r3 and r5 after 5s (Fig 3). These features suggest that they are involved in the maintenance of *krox20* expression and possibly in the underlying positive feedback loop [21]. In addition, the chick and mouse orthologues of element A contain Krox20 binding sites that are required for enhancer activity [20,21], and mouse element A is absolutely necessary for *krox20* autoregulation [21]. To investigate whether zebrafish elements A, D and E could be involved in direct *krox20* autoregulation, we first examined the activity of these elements in the absence of the Krox20 protein. As indicated above, without Krox20, the enhancer activities of elements D and E were completely abrogated in both r3 and r5 (Fig 5A), demonstrating that these elements are Krox20-dependent and are likely to be involved in the feedback loop. In the case of element A, in the absence of Krox20 protein, r3 enhancer activity was completely eliminated, but some r5 activity was maintained, although severely reduced (Fig 5A). These data indicate that element A possesses a dual function: Krox20-dependent enhancer activities in both r3 and r5 and a Krox20-independent enhancer activity specifically in r5. This latter activity is likely to contribute, together with elements B and C, to the initiation of *krox20* expression in r5 (Fig 4).

To determine whether the Krox20-dependent activities of elements A, D and E might involve direct binding of the Krox20 protein, we looked for potential binding sites for Krox20 within the enhancer sequences. For each, we identified several putative binding sites (S1 and S2 Figs). Oligonucleotides corresponding to sequences from each enhancer and carrying two of these binding sites were synthesized and used to perform gel retardation experiments in the presence of the Krox20 protein, together with specific or non-specific competitors. In each case, there was at least one strong retarded band, corresponding to a specific complex with Krox20 (S5 Fig), indicating that these elements contain high affinity Krox20 binding sites and supporting the idea that their enhancer activity is dependent on direct binding of Krox20.

As the phenotypes associated with the single homozygous mutation of elements A, D or E are limited, it is likely that these elements cooperate to establish full autoregulation. We tested this hypothesis by combining the different mutations. Indeed, the combination of two homozygous deletions, affecting A and D, A and E, or D and E severely reduced *krox20* expression at 12s and 22s (Fig 5B). Furthermore, elimination of the three enhancers, either by introduction of deletions affecting each one (Fig 5B) or by combination of a deletion of element A with a deletion of the D-E region (S2 and S4 Figs) led to complete loss of *krox20* expression at 12s and 22s. Note that neural crest expression at 12s, which relies on element B, is maintained in all cases (Fig 5B).

In conclusion, our data establish that elements A, D and E all carry Krox20-dependent enhancer activities. Furthermore, these elements cooperate to generate the positive feedback loop that maintains late expression of *krox20*. Finally, these activities are likely to involve direct binding of the Krox20 protein to each enhancer.

### *Cis*-dependent functional interactions between elements A and C

**Fig 6:**
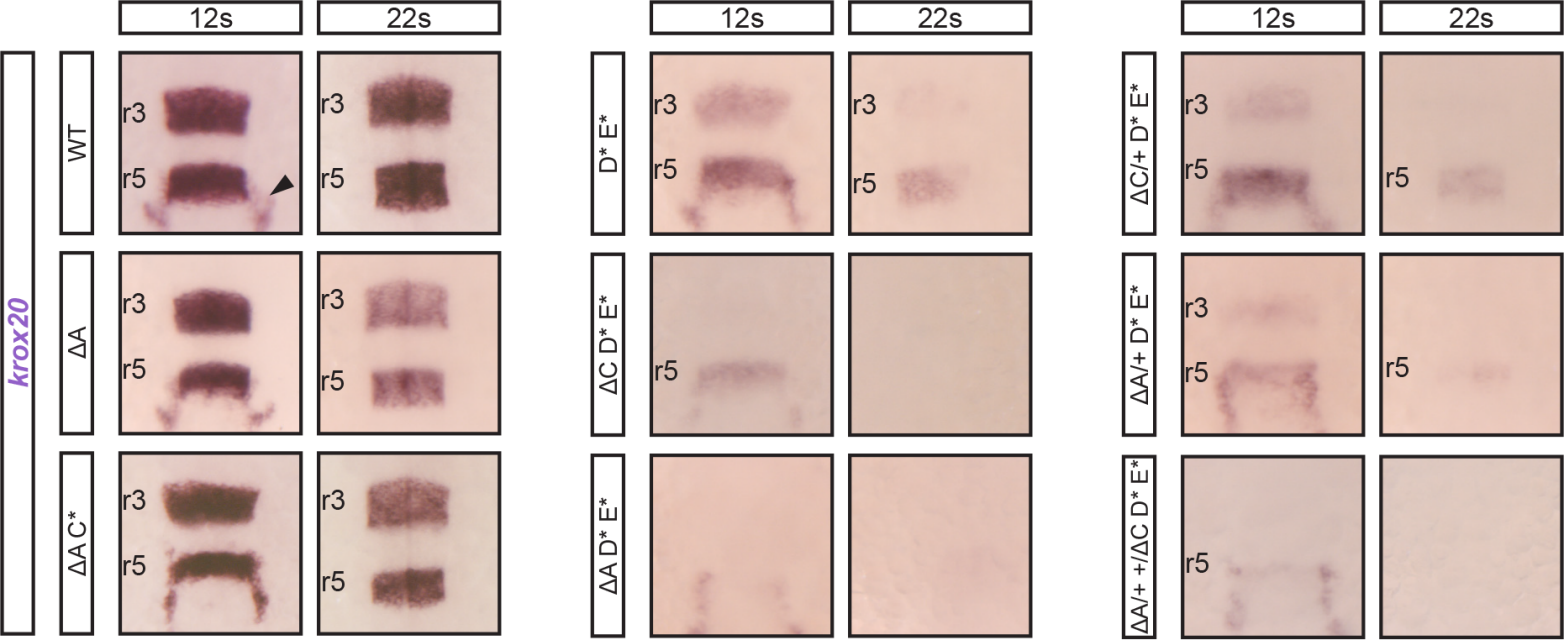
Collaboration in *cis* between elements A and C for the control of autoregulation. Embryos carrying combinations of homozygous deletions of elements A (∆A), C (C*), D (D*), E (E*) and of heterozygous deletions of elements A (∆A/+) or C (∆C/+) were analysed for *krox20* expression by in situ hybridization at the indicated stages. The genotype (∆A/+ +/∆C) corresponds to heterozygous deletions of A and C affecting different chromosomes. Somatic deletions are indicated by the * symbol and positions of r3 and r5 are shown. Neural crest cells migrating from r5/r6 are indicated by an arrowhead.

On the basis of the above analysis, element C appears somehow peculiar. Like elements A, D and E, it shows enhancer activity in r3 and r5, but this activity is Krox20-independent (Figs 1B and 5A). Furthermore, its activity is not restricted to r3 and r5, but also covers r4, with a dynamic anterior-posterior pattern (Fig 1B). Deletion experiments have shown that element C is a minor contributor to initiation of *krox20* expression in r5 (Figs 3 and 4). It is also involved in late *krox20* expression, as its deletion leads to a slight decrease in *krox20* mRNA levels in both r3 and r5 after 5s (Fig 3), although this is not likely to occur via direct autoregulation (Fig 5A). To determine whether element C interacts with other elements at late stages, we combined its deletion with mutations in A, D and E. Inactivation of element C did not exacerbate the late phenotype associated with the elimination of element A (Fig 6, compare ∆A and (∆A C*)). In contrast, the phenotype was more severe when mutation of element C was combined with mutations of elements D and E (Fig 6, compare (D* E*) and (∆C D* E*)). In fact, this latter genotype leads to a phenotype similar to that of (∆A D* E*), although slightly less severe in r5 (Fig 6), probably due to the more significant involvement of element A in the initiation of *krox20* in r5, as compared to element C. Together, these data are consistent with element C contributing to autoregulation by modulating the activity of element A.

Similar cooperation was previously observed in the mouse, where the orthologue of element C, although not directly participating in the positive feedback loop, cooperates in *cis* with element A to potentiate its autoregulatory activity [24]. To investigate whether such a *cis*-cooperation exists between A and C in zebrafish, we generated embryos homozygous for D and E mutations and heterozygous for A and/or C deletions (Fig 6). The latter were introduced by crossing ∆A and ∆C homozygous lines and were therefore present on different chromosomes. When both heterozygous deletions for A and C were present (∆A/+ +/∆C D* E*), *krox20* expression at late stages was affected in a manner similar to the combination (∆A D* E*), where the deletion of element A is homozygous. In contrast, when only the heterozygous deletion of C was introduced in the (D* E*) background (∆C/+ D* E*), it did not significantly increase the severity of the (D* E*) phenotype (Fig 6). These results support the existence of a *cis* interaction between C and A, required to allow A to participate in the autoregulatory loop.

Together, these data indicate that element A does not take part in autoregulation when a functional element C is not present on the same chromosome. Therefore, element C cooperates with element A to potentiate its autoregulatory activity, just as in the mouse. However, in the zebrafish, two additional *cis*-regulatory elements, D and E, directly participate in the feedback loop. In contrast to element A, element D and E are not likely to depend on element C to exert their enhancer activities.

### Plasticity of element A functions during vertebrate evolution

**Fig 7:**
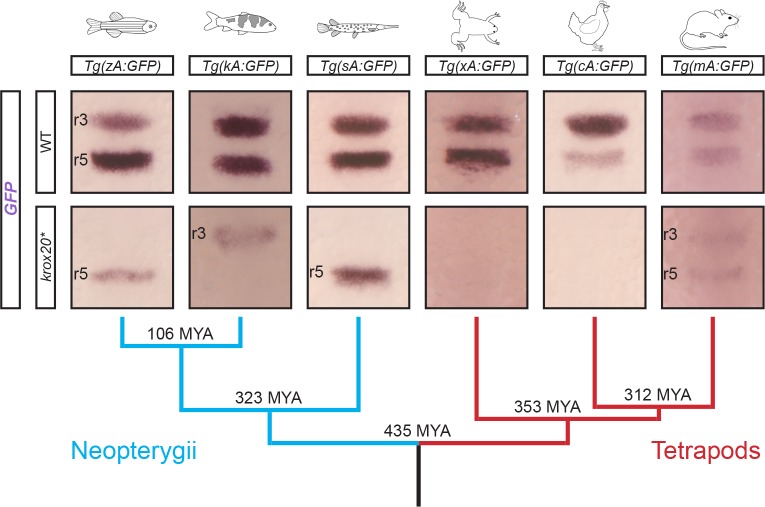
Evolution of enhancer A activity in vertebrates. The orthologues of element A from 6 vertebrate species, zebrafish (zA), koi carp (kA), spotted gar (sA), *Xenopus laevis* (xA), chicken (cA) and mouse (mA) were transferred into a GFP reporter construct and the corresponding plasmids were used to generate zebrafish transgenic lines, as indicated. *GFP* expression was analysed by in situ hybridization at 8s in embryos from each line, either in wild type (WT) or *krox20* null (krox20*) backgrounds, the latter being obtained by injection of Cas9 protein together with guide RNAs targeting the coding sequence of Krox20’s zinc fingers. Positions of r3 and r5 are shown. A phylogenetic tree with the indication of the node time distances from the present in millions of years (MYA) is shown underneath.

Zebrafish element A acts both as a Krox20-independent initiator element in r5 and as an autoregulatory element in r3 and r5. The existence of these dual activities is surprising in view of what we know of its chicken and mouse orthologues. Chicken element A is totally dependent on Krox20 binding for its enhancer activity, as demonstrated by comparison of a reporter transgene in mouse *Krox20* null and wild type backgrounds, and by mutation of element A Krox20 binding sites with enhancer activity assessed in chick embryos [20]. In addition, while deletion of mouse element A completely abolishes the positive feedback loop, it has no effect on early expression in r5 in this species [21]. Therefore, element A does not appear to act as an initiator element in chick nor mouse, suggesting its enhancer activity has been modified during vertebrate evolution. To investigate whether a coherent pattern of evolution of the element might be identified, we analysed the activities of orthologues of element A from several key species in the vertebrate phylogenetic tree (Fig 7). We cloned the orthologues of zebrafish element A (zA) identified by sequence alignments from koi carp *Cyprinus rubrofuscus* (kA), spotted gar (sA), *Xenopus tropicalis* (xA), chicken (cA) and mouse (mA) into the ZED *GFP* expression vector, generated stable zebrafish transgenic lines (at least two independent ones for each species) and determined the patterns of *GFP* expression by in situ hybridization. In a wild type zebrafish background, despite the heterospecific character of the assay, all elements behaved similarly and could direct *GFP* expression in r3 and r5, although there were some relative variations in the expression level between the two rhombomeres (Fig 7). To determine whether any of these enhancer activities were dependent on the Krox20 protein, we injected transgenic embryos from each line with the Cas9 protein and guide RNAs targeting the sequences encoding the three zinc fingers of the Krox20 protein, which constitute the DNA binding domain (S2 Fig). This treatment effectively abolishes *krox20* expression at 12s (S6 Fig), and allows the assessment of Krox20-independent enhancer activity. A large proportion of the activities of the enhancers was Krox20-dependent (Fig 7). However, limited Krox20-independent activities were maintained in some cases. Surprisingly, their patterns appeared different from one species to another and incoherent with the phylogenetic tree: the zebrafish and spotted gar elements remained active in r5 only, whereas the koi carp element was only active in r3; the mouse element was weakly active in both r3 and r5, whereas no activity was detected with the Xenopus and chick elements (Fig 7).

In conclusion, this analysis shows that the features required for Krox20-dependent expression of element A are likely to have been largely conserved during the course of vertebrate evolution. In contrast, the capacity of this element to combine its autoregulatory activity with Krox20-independent initiator functions appears highly contingent, with no clear correlation with the course of evolution. Furthermore, this Krox20-independent activity can occur in r3, in r5 or in r3 and r5, revealing a surprising plasticity of element A for acquiring and losing additional functions during evolution.

## Discussion

**Fig 8:**
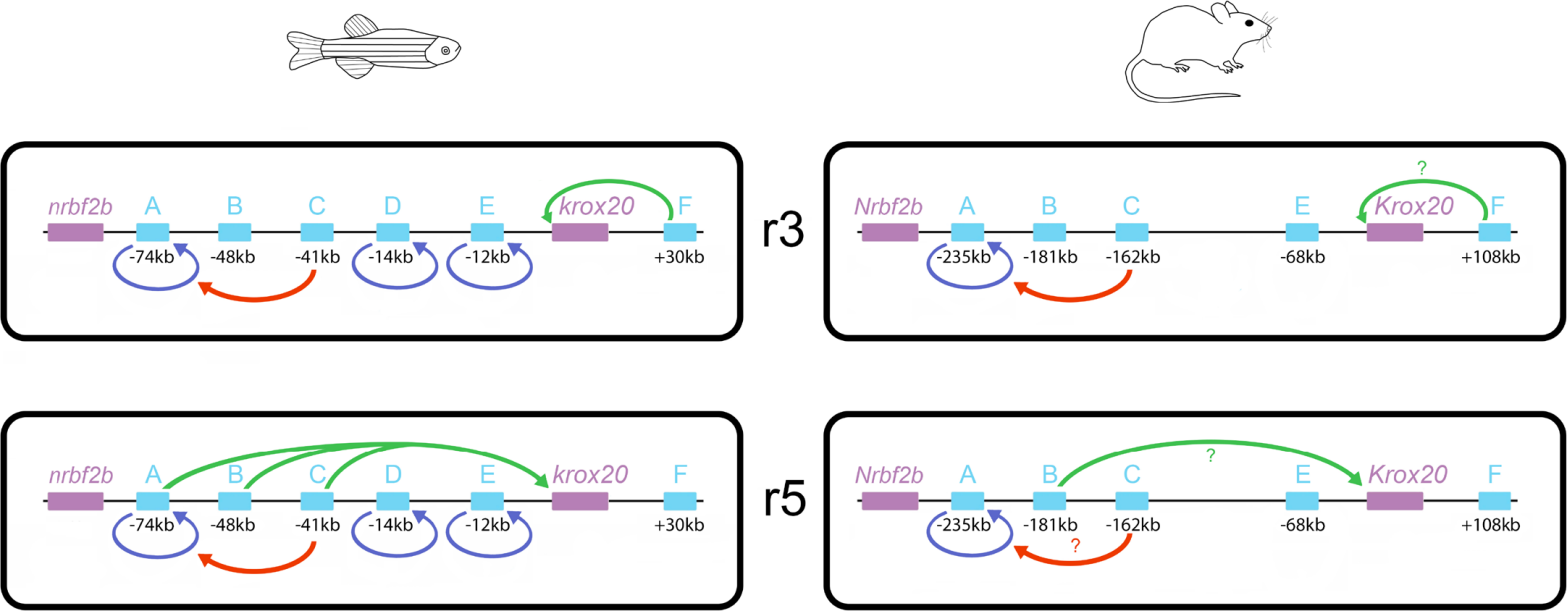
Schematic of the *cis*-regulation of *krox20* expression in r3 and r5, illustrating differences between zebrafish and mouse. *Cis*-acting elements are indicated by light blue boxes along the locus, with their position with respect to the site of transcription initiation underneath. The different types of activities of the elements are represented by arrows originating from the element: enhancer activities involved in the initiation of *krox20* expression are indicated by green arrows pointing toward the promoter, enhancer activities corresponding to direct autoregulation are indicated by blue arrows pointing back to the element and the potentiator activity of element C is represented by red arrows pointing toward element A. Question marks indicate that the activity is suspected, but not confirmed.

In this work, we have performed a comprehensive functional analysis of the *cis*-regulatory landscape of an important developmental gene, *krox20*. In the zebrafish, the organisation appears highly complex, since no less than 6 *cis*-acting elements are required to control the expression of the gene in two rhombomeres. These elements can account for all aspects of *krox20* expression in the developing hindbrain, allowing us to propose a global view of its regulation (Fig 8). As previously observed in the mouse, the enhancer activities of these elements can be classified as Krox20-independent or -dependent, the latter underlying the positive feedback loop that ensures amplification and maintenance of *krox20* expression at late stages. Apart from the initiation of *krox20* expression in r3, the other aspects of the regulation of the gene are controlled by multiple elements: initiation of *krox20* expression in r5 is governed by 3 elements (A, B and C), whereas autoregulation is controlled by 4 elements (A, C, D and E). These elements appear to cooperate according to various modes, possibly involving *cis*-interactions. Surprisingly, two elements (A and C) appear to participate in both regulatory aspects, revealing an intriguing interplay that might originate from the sharing of some binding sites for transcription factors involved in both activities. Finally, comparisons among vertebrates have revealed that the *krox20 cis*-regulatory landscape is unexpectedly poorly conserved and that particular elements show a remarkable evolutionary plasticity.

### Co-operation and redundancy of enhancer elements

Several studies, mostly performed in *Drosophila*, have recently shown that cooperation between *cis*-regulatory elements is a common feature in the regulation of developmental genes and can occur according to different modes, including in particular additive, synergistic or hierarchical interactions [3,5,24,29,30]. The present study provides examples of such co-operations in vertebrates, in the initiation of *krox20* expression in r5 as well as in the positive feedback loop (Fig 8). Although we have not performed quantitative analyses of the contributions of each *cis*-acting element to the different aspects of *krox20* hindbrain expression, in the case of initiation in r5, this cooperation appears to occur through an additive mode: deletion of each element leads to reduced expression (with B>A), and a drastic decrease requires combination of both deletions. The third element, C, appears only as a minor contributor to this activity. More generally, the transcriptional activity of each of these r5 initiating elements shows specificities (Fig 1B) that may reflect differences in which transcription factors act on them [20,22,23,31].

Considering autoregulation, three elements (A, D and E) can directly bind Krox20 protein (S5 Fig). Elimination of each one alone leads only to a mild phenotype (with E>A>D, Fig 3). However, combined knockdown of any two elements results in a major decrease in late expression (Fig 5B), suggesting the existence of a strong synergistic component in this co-operation. Therefore, in this case, synergy and redundancy are not exclusive, as an almost full activity is already reached with two elements.

Redundancy in the *cis*-acting elements controlling zebrafish *krox20* autoregulation differs remarkably from the situation in the mouse, in which element A is absolutely required for late *Krox20* expression [21]. While we were not able to detect sequences homologous to element D in the mouse *Krox20* locus, there is a poorly conserved mouse orthologue of element E, although, it cannot rescue the deletion of element A in this species. Overlapping activities between regulatory elements add robustness to the expression of developmental genes [3,32]. We speculate that the difference in redundancy in the control of the *krox20* feedback loop between zebrafish and mouse might reflect differences in both external and internal conditions that require additional robustness in the zebrafish. For example, the zebrafish embryo is much more sensitive to modifications in environmental conditions such as temperature or mechanical stress. Further, the process of hindbrain segmentation takes only 12 hours in the zebrafish compared to 36 hours in the mouse, giving the zebrafish much less time to ensure full establishment of *krox20* autoregulation, a crucial step in building normal size r3 and r5 [21].

### Cooperation in *cis* and chromatin organisation

The additional involvement of element C in autoregulation is peculiar, as it seems to operate in a *cis*-acting, hierarchical manner with element A. In contrast, element C does not potentiate the autoregulatory activities of elements D and E, since in the absence of element A, elimination of element C does not affect autoregulation. It is possible that this independence of elements D and E from C might be related to the organisation of the locus itself, given that elements D and E are much closer (-15 kb, -12kb) to the promoter than element A (-74 kb), with element C being positioned in between (-41 kb). Interestingly, the potentiation by C is only required for the autoregulatory activity of element A, but not for its initiator activity in r5. Therefore, if element C is required for chromatin opening at element A as proposed in the mouse [24], the constraints on chromatin structure for activation by the Krox20 protein are likely to be different from those required by the initiation factors.

It is worth noting that element F, which is in charge of the initial activation of *krox20* in r3, the earliest manifestation of *krox20* expression in the embryo, is the only hindbrain regulatory element to be located downstream of the gene, whereas the elements responsible for initiation in r5 and autoregulation are all located upstream. We speculate that this spatial organisation might reflect the existence of two mutually exclusive DNA loops, as observed for the regulation of the *HoxD* cluster during vertebrate limb development [33]. Early in r3, a DNA loop might form, including the *krox20* promoter and the downstream region containing element F. Later, the promoter might engage into an alternative loop including all upstream elements, allowing initiation of *krox20* expression in r5, as well as establishment of autoregulation in both rhombomeres. This dynamic spatial organisation is consistent with the very early activation of element F (Fig 1) and premature downregulation of *krox20* in r3 in the ∆(A-E) mutant (Fig 3). Later, this organisation would allow parallel activation of elements involved in initiation in r5 (A, B and C) and autoregulation (A, C, D, and E), with two elements (A and C) participating in both processes.

### Versatility of *cis*-regulatory elements

It is surprising that some *cis*-acting regulatory elements have the capacity for different activities that might have been expected to be carried out by distinct elements. We found two examples of such versatility. The first is zebrafish element A, which possesses two distinct types of enhancer activities: a Krox20-independent initiator activity in r5 and a Krox20-dependent autoregulatory activity in both r3 and r5. The second case, element C, is even more striking. This sequence appears to carry enhancer activity in r3-r5 when assayed in the transgenic reporter system and it contributes in vivo to the initiation of *krox20* expression in r5, presumably via this classical enhancer activity. In addition, element C appears to also function through cooperation in *cis* with element A, to potentiate its autoregulatory activity in r3 and r5. We have previously proposed, in the case of mouse element C, that such a potentiating activity, required for the function of a positive feedback loop, may constitute an efficient safety lock against inappropriate activation of autoregulatory elements [24].

At this stage, in the absence of analyses of the precise DNA sequences required for the activities carried by A or C, it is not known whether the dual functions are borne by distinct sequences or involve some common sequence motifs and interacting factors. In the former case, we would expect adjacent or intermingled *cis*-acting elements. The latter possibility is more interesting, as the sharing of some binding sites might result in common properties, like temporal and/or regional domains of activity. Hence, element C enhancer and potentiator activities overlap in r3 and r5 between 3s and 10s. Preliminary efforts designed to separate initiating and autoregulatory activities of element A by external deletions have failed, both activities decreasing in parallel. Finally, composite organisation underlying different activities might facilitate the appearance and modifications of one activity by mutations, leading to increased potential for evolution.

### Evolutionary plasticity of *krox20 cis*-regulation

Comparison of the *krox20 cis*-regulatory landscape between zebrafish and mouse revealed major differences in the number of elements, in their nucleotide sequences and in their functional activities (Figs 2 and 8). This is particularly surprising in view of the strong conservation of hindbrain segmentation and the *krox20* expression pattern during vertebrate evolution, and given that modification of *cis*-regulatory sequences is considered a major driver of evolution in higher organisms [7]. Among the 6 *cis*-regulatory elements identified in zebrafish, only two are relatively strongly conserved among vertebrates–elements B and F (S1 Fig), which are the only ones involved exclusively in the initiation of *krox20* expression (Fig 2). In contrast, among direct autoregulatory elements, elements A and E are poorly conserved between zebrafish and mouse (S1 Fig), and element D is not present in tetrapods, but instead detected cavefish. This correlation between initiation versus autoregulation and evolutionary conservation might be explained by the need for initiating elements to act as platforms integrating numerous signals mediated by a variety of transcription factors, to precisely define spatial and temporal domain of activity. This platform function might seriously constrain the evolution of enhancer sequences. In contrast, direct autoregulatory elements mainly need to bind the gene product, probably as well as factors that more loosely restrict the domain of autoregulation. This is likely to offer additional evolutionary plasticity. Multiplication of partially redundant elements, like in the case of autoregulation, also offers space for increased evolutionary flexibility. In contrast, elements F and B play unique or major roles in r3 and r5 initiation, respectively, and are therefore likely to be more constrained.

The search for putative binding sites for transcription factors likely to control the regulation of *krox20* expression supports this interpretation. Hence, vHnf1 and MafB binding sites, and Hox/Pbx, Meis and Sp binding sites are well conserved between mouse and zebrafish elements B and C, respectively (S1 Fig). These different factors and their binding sites have been shown to play essential roles for the enhancer activities of the corresponding chick and mouse elements (Chomette et al., 2006; Wassef et al., 2008 and Labalette et al., 2015), suggesting similar functions in the zebrafish. Furthermore, like element C, element F contains conserved binding sites for Hox/Pbx, Meis and Sp factors, suggesting that the two elements bind overlapping subsets of transcription factors, and that these common factors might be essential for element F activity in r3. In this respect, it is worth noting that elimination of the Meis sites in chicken element C have been shown to affect its enhancer activity specifically in this rhombomere in transgenic mice (Wassef et al., 2008). Meis factors might therefore play a particularly important role for element F.

Another interesting evolutionary issue is the case of enhancers possessing dual activities. The zebrafish autoregulatory element A carries an additional, Krox20-independent activity in r5, in contrast to its chicken orthologue. The appearance of this additional activity might have been favoured by the redundancy in the elements governing autoregulation in the zebrafish. In any case, we explored the presence of dual activities in element A in several vertebrate species to determine whether this would correlate with the phylogenetic tree. However, the activity patterns were unexpectedly variable, with no correlation with evolution (Fig 7): the Krox20-independent activity, as tested in the zebrafish, can be restricted to r5 (zebrafish and spotted gar), to r3 (koi carp), present in r3 and r5 (mouse) or absent from the hindbrain (Xenopus, chicken). To determine whether the pattern of Krox20-independent activity of the elements A might correlate with the presence of binding sites for specific transcription factors, we searched for putative binding sites known to be involved in the r3- or r5-specific activities of elements B and C. Zebrafish element A contains several MafB sites and a single vHNF1 site. Whereas MafB putative sites were observed in the carp element, no vHNF1 site was found. As this binding site is essential for the r5-specific activity of element B, this might explain the absence of initiator activity of the koi carp element in r5. In contrast, concerning the r3-specific expression of the carp element, the distribution of putative Hox/Pbx and Meis sites does not provide any clue susceptible to explain the different behaviour of the two elements. In any case, our analysis suggests that element A shows a high potential and plasticity for developing initiation functions, possibly favouring adaptation to various embryonic environments. It will be interesting to determine whether this plasticity is linked to the dual nature of the element and whether this feature has a broad significance. Indeed, it has been proposed that evolution of novel patterns of gene expression relies on the introduction of mutations in pre-existing enhancers rather than on the invention of new ones [34,35]. In this respect, element A might have been caught red-handed.

## Materials and methods

### Ethics

All animal experiments were performed in accordance with the guidelines of the Council of European Union Directive n°2010/63/UE and were approved by the “Comité d’éthique pour l’expérimentation animale Charles Darwin” (Project Number: APAFIS#848-2015061510065446v3).

### Constructs, transgenic zebrafish lines and in situ hybridization

The constructs used to generate transgenic zebrafish lines were based on the ZED plasmid [27] digested by BspM1+BspEI, in which each of the tested regulatory elements were cloned upstream of a *GFP* reporter gene driven by the *gata2* minimal promoter, using Clontech’s “In-Fusion HD Cloning Kits” and following their protocol. The DNA primers used to amplify the regulatory elements were designed with the forward (5’-TGAATGCTCATCCGGA…-3’) and reverse (5’-GACCTGCAGACTGG…-3’) prefixes complementary to the ends of the linearized vector, which were followed by the specific sequences of the primers (S1 Table). Primers were synthesized and purified by Eurofins Genomics. Transgenic lines were obtained from embryos injected at the one-cell stage with 50 pg of Tol2 transposase mRNA [36] together with 75 ng of the ZED construct. At least two independent transgenic lines have been generated and analyzed for elements A, B, C, D and E and the elements A from various species. Single and double whole-mount *in situ* hybridizations were performed as described [37], with the previously published digoxigenin-labelled riboprobes for *krox20* [38] and *GFP* [31].

### CRISPR/Cas9 RNA guide design and injection

Zebrafish lines with regulatory elements deleted, embryos harbouring somatic deletions of one or several enhancers and embryos with somatic deletions of the zinc finger domain of *Egr2b* were generated using the CRISPR/Cas9 editing system. The sequence-specific parts of the RNA guides (S1 Table) were designed with the help of the CRISPOR design tool (http://crispor.tefor.net/) to minimise off-targeting, maximise efficiency and specificity of targeting and, in the case of somatic deletions, to target putative binding sites or particularly conserved regions within enhancers (S1 and S2 Figs). Four guides were designed to target element A on two Krox20 binding sites and two conserved regions. Four guides were designed to target element C on two putative binding sites for Hox/Pbx factors, one for Meis and one for Sp. Four guides were designed to target element D on four binding sites for Krox20. Three guides were designed to target element E on three binding sites for Krox20. Three guides were designed to target element F on one putative binding site for Hox/Pbx factors, one for putative Meis binding site and one conserved regions. The targeting parts of the RNA guides were synthesized (Integrated DNA Technologies) 5’ to a 15-nucleotide sequence complementary to the tracrRNA 5’-GUUUUAGAGCUAUGCU-3’. This “crRNA” was then hybridized with the 67-nucleotide long “tracrRNA” to form the complete RNA guide. These complete guides (50 μM) were then incubated with the Cas9 protein (45 μM) (synthesized and generously provided by Anne de Cian, Muséum National d’Histoire Naturelle) in the Cas9 buffer (20 mM Hepes pH 7.5, 150mM KCl) and injected into one-cell stage zebrafish embryos. Founder zebrafish for knockout lines and whole injected F0 embryos harbouring somatic deletions were genotyped after the *in situ* hybridization analysis using PCR genotyping primers (S1 Table). Sanger sequencing was performed (Eurofins Genomics) to characterise the deletions obtained through non-homologous end joining.

### ATAC-seq

ATAC experiments were performed according to Buenrostro and colleagues [26], using a homemade transposome [39]. All embryos were dissected in cold PBS to remove the vitellus (50 embryos at 95% epiboly) or to isolate the hindbrain and the posterior part of the embryo (80 embryos at 5s and 50 embryos at 15s) as shown in Fig 2. Biological duplicates were performed for each ATAC experiment. Briefly, cells were lysed before transposition using 1 μl of transposome and purified using a Qiagen MinElute Kit with 10 μl of Elution Buffer. Transposed DNA was amplified by PCR [39] and quantified by qPCR as previously described [24]. Sequencing was performed on multiplexed samples using 42 bp paired-end reads on an Illumina NextSeq according to the manufacturer's specifications. For computational analysis, paired-end reads were mapped onto the zebrafish genome assembly zv9, using STAR as previously detailed [24].

### Protein extracts and gel shift assays

The mouse Krox20 protein was expressed in bacteria using the pet3a system. Extracts were prepared from *Krox20*-expressing and control bacteria as previously described [40]. Double-stranded biotinylated oligonucleotides with the following sequences were used as probes:

Oligo A:
5-’CCAGGTTCTCCTGTGTGCTTTTGGAGCGCTTGTATGGGGAATCCCACATATCCT
CCTACACACAGCGCCC-3’;
Oligo D:
5’-CAATTGTTCTTTTAACCGAGTAGGAGGCCGTTTTTCTTAGTGATTTTGTGTAGG
TGTTACTTTTCTATAG-3’;
Oligo E:
5’-GCATCTGTAGGCGTACAACAAAAGACACATGCATCTGAAGAAGTTTCCTTCTT
TCATTTGTGGGGGAGGACGC-3’.
Double stranded oligonucleotides with the following sequences were used as competitors:
WT: 5’-CTCTGTACGCGGGGGCGGTTA-3’;
MUT: 5’-CTCTGTACGCGG**C**GGCGGTTA-3’.

Gel shift experiments were performed with the light shift chemoluminescent EMSA kit (PIERCE), except for the composition of the binding buffer [40].

### Accession codes

The data have been deposited in the Gene Expression Omnibus (GEO) under accession number GSE113471 and are available at the following address: https://www.ncbi.nlm.nih.gov/geo/query/acc.cgi?acc=GSE113471

## Supporting information

S1 Fig Sequence alignments of enhancers between different species and search for putative transcription factor binding sites. The first panels show sequence alignments of zebrafish and mouse entire element A and core regions from elements B, C, E and F. The non-conserved element D sequence is also shown. The last panel shows a sequence alignment of zebrafish and koi carp element A. Conserved nucleotides are marked by a star, and characterized or putative binding sites for transcription factors are indicated: Krox20 (K20) in red, Mafb in blue, vHnf1 in green, Hox/Pbx (HP) in purple, Meis in pink, and Sp in orange.
(PDF)

S2 Fig Sequences of the different regions of the zebrafish *krox20* locus in which deletions were introduced. Each panel shows the entire (A to F and *krox20*) or partial, (D-E) and (A-E), nucleotide sequence of the considered region. The DNA fragments with enhancer activity that were used to drive GFP expression in transgenic lines are shown in green. The target sequences for the guide RNAs used to generate the germline deletions are shown in red and the sequences eliminated in those deletions are indicated by capital letters. The target sequences for the guide RNAs used to generate the somatic deletions are shown in blue. Putative Krox20 binding sites in elements A, D and E are underlined (pink). The sequence encoding Krox20 zinc fingers is underlined (black) and the single nucleotide mutated (G to A) in *krox20*^*fh227/fh227*^ is shown in orange. Diagrams above the sequences indicate the approximate positions of the targeted sequences within the locus.
(PDF)

S3 Fig Analysis of somatic deletions. (A) PCR amplification of either wild type (WT) embryos or F0 injected embryos (MUT), using the primers indicated in S1 Table. F0 embryos were injected at the one-cell stage with CRISPR/Cas9 ribonucleic complexes targeting elements A, C, D, E, F or the *krox20* gene and PCR amplifications were performed on whole embryos at stages 12s (for element A, and the *krox20* gene) or at 22s (for elements C, D, E and F), corresponding to stages of *in situ* hybridization analysis. Complete elimination of the wild type diagnostic fragments demonstrates the high efficiency of the procedure. (B) Sequence alignments of wild type element C (WT) with five mutated alleles generated by CRISPR/Cas9 injections. Red brackets indicate the limits of the deletions and putative binding sites for transcription factors are indicated: Hox/Pbx (HP) in purple, Meis in pink, and Sp in orange.
(PDF)

S4 Fig Very similar phenotypes obtained following germ-line or somatic deletions. Embryos from three lines carrying homozygous deletions of a *cis*-acting element (∆) or F0 embryos carrying somatic mutations (*) in the same elements were analysed for the expression of *krox20* by in situ hybridization at the indicated stages. WT, wild type control. Positions of r3 and r5 are shown.
(PDF)

S5 Fig Presence of Krox20 binding sites within enhancers A, D and E. Oligonucleotides corresponding to sequences present in enhancers A, D and E and covering Krox20 binding sites (see the Materials and Methods section for the sequences of the oligonucleotides and S2 Fig for the positions of the Krox20 binding sites) were subjected to polyacrylamide gel retardation assays. Biotin-double-stranded oligonucleotides were exposed to bacterial (Pet) extracts containing the Krox20 protein or not (-), in the presence or absence (-) of an unlabelled oligonucleotide competitor. The competitor oligonucleotides carried either a bona fide Krox20 binding site (WT) or a mutated version (Mut) that does not allow Krox20 binding. The arrows indicate the migration positions on the gel of the free oligonucleotides (free probe) and of retarded bands corresponding to specific complexes with the Krox20 protein.
(PDF)

S6 Fig Loss of Krox20 activity in embryos injected with guide RNAs targeting sequences encoding Krox20’s zinc fingers. Transgenic 12s embryos carrying the GFP reporter driven by the A enhancer were either uninjected (Control) or injected with Cas9 and guide RNAs targeting the coding sequence of Krox20’s zinc fingers (krox20*) and analysed by single in situ hybridization with a *krox20* probe (purple labelling) or double in situ hybridization with *krox20* (purple labelling) and *GFP* (orange labelling) probes.
(PDF)

S1 Table Sequences of the 3’ end of the cloning primers used for constructing the ZED constructs, the 5’ end of CRISPR/Cas9 RNA guides used to create the germ-line and somatic deletions and the PCR primers used to detect the deletions. (PDF)
