# Supplementary material for: Cooperation, cis-interactions, versatility and evolutionary plasticity of multiple cis-acting elements underlie krox20 hindbrain regulation
Source: PLoS Genet. 2018 Aug 6;14(8):e1007581. doi: 10.1371/journal.pgen.1007581 (PMC6095606; doi:10.1371/journal.pgen.1007581)
Supplement: S1 Fig — The first panels show sequence alignments of zebrafish and mouse entire element A and core regions from elements B, C, E and F. The non-conserved element D sequence is also shown. The last panel shows a sequence alignment of zebrafish and koi carp element A. Conserved nucleotides are marked by a star, and characterized or putative binding sites for transcription factors are indicated: Krox20 (K20) in red, Mafb in blue, vHnf1 in green, Hox/Pbx (HP) in purple, Meis in pink, and Sp in orange. (PDF) [file pgen.1007581.s001.pdf]

Alignment zebrafish-mouse element A

```
                                                                                                                                                  K20
A_zebra     ----------------------------------------GTAGCGTAGTAGGCCCAGTAAAGCAGC---TACAACTCTGCACAGTTTAAAAAACGTGGCAAATTACCTC--------AG
A_mouse     CCAAAAGCAGGCCTTCTCTGTCAGAGATTTCTGATTCACGTCTCCCCTCAGTCAGAAGACAGCAGCAGCAGCCAGTGACAGAAGTCTCCTGACTGACATCTATCTGCTGTGTGGGTGAA
                                                    **  *     ** *  *  * ******     * * *      *** *    *  *       **   **         *

A_zebra     AATATAG--GCTATATGTCA-----TATATGGCATATTATATACATCATTTTTGATACATTTTAAAATGTGTGTAGCATGTA---------CAAAACAT---------------------
A_mouse     CTGGTACCCGCCCTGACTGCTGCCTTGACTACTGTGTTTCAGAGAGCAGGCTTTCTGAACCATGGGTTATCTGAAGGAGCTGACGATGATGCAAGAGACACGGCCTTAGTAGCGAGCCCG
                **   **  *   *       *   *    * **  * * * **   **  *  * * * * ** ** *  *          *** * *

A_zebra     ----------------------ATTTCATTCTT----CCACTTACCACTAGAAGGAAGCCTGCGTACCACAGTTTGAGAACCACTGCTCTAGAGTAATTTTCAAGACACGATTCCAATCC
A_mouse     GCCTCCTCTAGTTGTCAACCACGCTCAATGTTTTCACAGATTCATCGCTACATCCTTGCAAGCCGACCAAACTCCGCCA-----TGCCTTGAAACCAGAGGCCAGAGATGCTGCAAAGTC
                                  *  **  **      * * * * *** *     **  **  **** * *  *  *     ***  *  *   *    * *** * * * * * ** *
                                       K20                                                                                           K20
A_zebra     ACTCCAAACCAGTTTCTGTGTGAAAGCCTCACACCTGACAGCGGTG--AGATCTGTCTGCTAGTTTAAC---------ATGTGTAAGAATATTTGTCAGGCACATTAGCTCACCGCAGGG
A_mouse     TC--ACTCCCTTATTATCTGCCAGTGCCTGGGACCTACAAATAGACATTCGTCTGTCTGTGAGTTCTGTATAGAGTGGTGGGAAAGGAAGAATGTGCAGGCACACAGGCACATGGGGGAA
             *      **   ** * **  *  ****   ****   *   *       ********  ****                   *   * *** * *   ********   ** **  *  *
                                                                                          K20          K20                          K20
A_zebra     TTCCCATTGTGTCCCCCGAGGAGGCCTTAAAAAATCCCCCCTCACATCCCCGTTGGCTCTTTTAGGGCGCTGTGTGTAGGAGGATATGTGGGATTCCCCATACAAGCGCTCCAAAAGCAC
A_mouse     TCCCTTTTGTCTCCCCACTGC-TTCTTCAAAGGATTCAGCATC-----TCTGACTCCCTTTGAGGACATCCCTGTGTGGGTGGCTCTGTGGGTCTCCCTGCAATGACaaaaaaaaaaaa
            * **  **** *****   *    * * ***  ** *  * **       * *    *  **   *    *  ***** ** ** * ******  ****   *    *    ****  *

A_zebra     ACAGGAGAACCTGGACAGAAGACCCGCTGTAAGCAGAATGTCTGGAGTCGGGACTCTCCTCTAT-AGCTTGGCTGGCTCATCATTACTTTTGAGGGCCGA-------ATCGAGGCAGGTG
A_mouse     aaaaaaaaaaaaaaaaaaa------aaaGCAAAGCAAAGCCCTGGATTGGTGCTTCCCTCAAAATGCAGAGTCTGGATTTTAAAAAAAGATAGGGATACAAAGCATGCTCAGGGAAGTCA
            * *  * **     * * *      * **    **   ***** * * *  ** *    *       * **** *  * *  *    *  **    *          **  ** **
                                K20    K20         K20
A_zebra     GAAGAGGCGACGGC--TCGTCCTCCTACACGCAGATTCCAGTGGA--CGTCA----AG-CTCTCGTTTTCTCACAGCTGTCGACTGCTGATGTGCTGACAGAAGAAGAGCTGG--AGATG
A_mouse     GAGCGCGGAGCAGAGTGTGGGCTGAGGAATGCCGCCTCCTCAGGGGGCGACAGTCAAAAGCGCTGGCTCATCGCGGGTGTCAAAGGCCGAGTTCTGCAGGTAAATAGCAATGAGATGCTT
            **    *   * *    *  **     * ** *  ***    **   ** **    *        *  *  ** * * **** *  ** **  *    *   **  **    **    * *
                 K20
A_zebra     AACACACACACACACACAGGAGAGAAGAATAGCTTAGTACTGACTTGATGCTGTGCGCTCAAATGATTTTAGCTGCTTGTCGAAATTACTTATTTAAAATGAGCT---------------
A_mouse     CCCTCCCACTCAGATTCCACACTGGAAGCTCGGGTATTGCT--------GGAGCCGCATCCACACCGCTGGCTCCATTCACAACCCCGCTCCTTTCAAGGAAACTCGCAGGCCTCTTAAA
              * * *** ** *   *    *   * *    * *   ** * **        *  *      ** *       *         **  * *      **  *** **    * **
                                                                   K20                                              K20
A_zebra     ------------------------------GAAACAACACA-------ATTCTGGAGGTGTTTTTGGTGGAGACCTAATTGTTTTACTCTGCATTTTTATACTGTGTGCTGCACTCTAAC
A_mouse     TGTGGCGGCCGGCCCCAGCCCAGAGCGGTTGCAGCAACAGACACAGGAAAGCTGTCACTTTTCTGGCTCCCAGCACTGACACTTATGTCGTCCTTTGTTTTCTGT------------GAC
                                          * * ***** *       *   ***     *** * * *       *         **     **   * *** * * ****                **
                                                                                           K20
A_zebra     ATAATCATTAAGTAAGTTAACTATTTTTTTCCAATTTAAATGGATTAAACTTTTTAAAAAAATAAGTTGTCCCCCGCCCCCAACACCCCCCCCCCTCTCTCAAAAAAAAACAAGAAATGT
A_mouse     ATGTCAGTTATCCAACAGC--CATTACTGCATCAACTGCAGGGACTACATATTTGTTATAAGCAAAACCTCGTTGAAGCCAACCCACCCCCCAGCCCTCCC-------------------
            **     ***   **        ***   *      *  *  * *** ** *  ***   * **  **    **         ** * *  ******  * *** *
```

Alignment zebrafish-mouse core element B

```
B_zebra CCCCAGTCCATGAATGTCCCCAATACAGTCCAAAACTACAGACAGGGCA-----ATTTTCTCTGACGTGCTCTGCCATCTTGTTTTGCACAGCGGGACAAAGGAGGTCCAAATGATGCCT
B_mouse CAGGAAAAAGAGGAAATTATAGCAGACACTAGAAAGTCCACACGCGGCACCTCCGCCCATCTCCCTACATTTCCACGTCCTGTGCAGCTTCTCTAACAATGAGTTGAAG-ATTGATGCTT
        *   *      * *  *               *** * ** **  ****                     *       ** ***   **    *      *             * ****** *
                                                      Mafb                                                 Mafb       vHnf1
B_zebra TTGTTGGAGCAGACGAACCCTTGACAGACGAGCTCCAGATCCAAAGCCTGCTCTTCCTGCTCTGCTTTGGCCG------------------------------GTGGGTTAATCTTCAACC
B_mouse TCAGGGATAATGAAGCCTGGAGCCACCTTAAAGACGTACAGATTAGTTGC-CGTTCCTTCTCTGCATGGGCTGAGAGATGGGAGAAAAATCAGCAGATTTCTGTGTGTTAATGTTCAACC
        *    *    **                  *   *      *       ***** ****** * *** *                                 *** ****** *******
                                 Mafb
B_zebra AGGTACAGGAACTGACCGTTGTGCTTACGTAGTGGAAGTGTGTGTCCATCCGTCTCCCCTGACAGAAAGCTCCACTTAGCTTGTCCTGGGCTTGTAACGCGCCATGTCCAATTAGCTCTC
B_mouse AGTACAGTGACGGGTATAAA--GCACAGGGCCGACAAGAGGGGCTTGACAGGCCAGCCCTTCCAGCCTATTGAAGTCGGCCTGTACTTGCC--GTAAGTCCCCTTGTCTAATTAGCTACT
        ***     **   *        **          *** * *  *  *   * *  ****  ***      *  * *  ** *** ** * *  ****  * ** **** ********

B_zebra GGGGTGCTCCGTCTTCATCC----------------CTAAAGACTGGAGAAAGCGCGTGGACCATGATGGAGGAGGCCTTGATGGGACAACAAGACGTCCTGCAGCCGCAGTCCACTGAG
B_mouse GAGCTGTTCCCTGGCTCCCTGCGTCTGACGGAGACTTCCTCGGGAAAAGTCTTGTCACCAAGCATGATGAAGTAGGGCTTGATTGTGCTACAAGATGTCCTGTAGATGCAGGCCACTTCT
        * * ** *** *     *                      *     **       *  * ******* ** *** ****** *  * ****** ****** **   **** *****
            Mafb                 Mafb                 Mafb
B_zebra TCGGCTAATCATCTACTGCATGCTCAGCTCTGGAGGTGAAAGTCTGTGGCCCAG
B_mouse GAGACTAATCATCAACTTTGCAGATTCCAGCGCCAGGGCTCATCTACAGGCCAG
          * ********* ***          *    *   * *    ***   * ****
```

Alignment zebrafish-mouse core element C

```
C_zebra --GCAGAGCTGTTGTCCAGGGGTGAAGACACAAAGGCCCAGTGGCAGGTCTTTTCTCCCCCAAAGCCGATACCACCTGCAGGGCCCGGGCC-TCTCCTGAGTGTCCCAGCACCACTCTCC
C_mouse ATGGCAGCCTTGCCTCCTAGGACAAAGCCCCAATAGCAAGCTGATACCACTTTTGACTAATCCGAGGGAAGCCACCTGCTGGAACTGTGTCTTTTCTGTGGGCTCCCTGCAGAACTTTCC
          *     **    ***  **    *** * ***  **     **  *    *****  *          **  ******** **  * * * * * * **     *  **** ***  *** ***
            HP                                   Meis            Meis                                                      Sp
C_zebra AAATGATGATTAATCACGGAGTGCTAAGAGGGGGGGGGTGGTTGCACCCTGACA----CA-GCCATAATGCTGGTCACCAGAGTCTGGATTGGCCAGCGGTGGTCAGTGGGTGGATGTCC
C_mouse AAATGATGATTAATCACTGCATAAATGGCAGCATTGACAACCACATAA-TGACAGAGAGAAAGCTGAGACCAGGGGCTCAACCGTGGGTGCTTGCCGCCCAGTCCCGTGGGTGGAGAAGC
        **************** *  *     *  *    *                *****     *   *   *   * **    **       **       * **   *  * *********    *
            HP                Meis
C_zebra GAGGAAGATGGATGACCTCGACCT-CCTGTCATTAACAAGAGAA-----------GAAACAAAACAGACAACAAAAAGAAAGGCAGCACGGTGTGAGGGAGAGTTTGTGTGGGAATAAAT
C_mouse CCAGAAGATGGATGGCTCGCAAGTGTCTGACACTTGAGTGTCTTGTCTTTTTACAAGGGTGGCCACTTCTACCCAATTCAACAAACTGGGCTGCGAGCAGCACATGGACTGGACTCACTT
           *********** *    *  *  *** ** *                                * **  **   **   *    * ** ***    *  * *  ***    *  *
                Meis                                 Meis     Meis
C_zebra GAGAGTTTTGACATTAGCGAACGTAAAGTAAACGCAAAGTAGTCCCTGACACGACATGACAGCGCTGAACTTGTGGAAGTTTGAGGATCGTTTGCTCTCATCAGCGGAAAGAGGAGCCGA
C_mouse CCTGTGGCTGTCAGAAGCAAGGAGAAAAATGCCTCAAA-------------CAAACAAACAAACAGGTTCCAATTGGCATCTTAAATCTAATG--------GCAACAGGAATGAGAGCC
                ** **  *** *    ***     * ****                *    ***     *  *   * *   * * * *    **               *   * ***
                Meis                                                                                                     HP
C_zebra GGTTTCTCTGACAGTTACAAATGAAGCCGCGATTCATTATTTTCCAGGGAATTCGCTCCTGCGCAGACGAAGATGGAGATGCAGCCGTGGCGTCACTGCCCATTTGGATGGAGATGAATC
C_mouse ACTGGCTGCAGATGGCAGCTTCCTCACTGTAGTGGAAGGCTTTAGGAAGGTTTCGCTGATTAGCA------CTTCAGATTACCCTAACGTTGTCCCTACTAATTTGAAAAATGATGTATC
          *  **     *  *         * *   *  *    ***     *  ******  *  ***          *     * *       *  *** ** *  ***** *    **** ***
                          Meis
C_zebra AAGGCGCTTTAGCCCAGACGCAGCACTGACAATCACATCAGCGAGGCTATCGGACTCCCCTCTACTCCTGGTGGACGGATCCGGTGCGGAGCGGAGCGGGTAAT------------AAAG
C_mouse ACTGAGATCAGACAGGGCTCCGAAGAGTCAATTACACTCGGCAGCAATATCAAAAGCTCATCTCTTCGACACCGAGGAATAAGACACGTTCTGCTTAATAACAAAAAAAAAAAAAAAAAA
        *  * * *    *   *   *          * *    ** **     ****  *  * * ***  **       ** * **  *    **    *          *             ***

C_zebra GCTCCTACTGAAACCCCATTAAGCCCGGTCTCCAAAGACCAGCTCATTAGCATAGCGGAATATTCCCCCCGTTTGTGTAATTCTATCAACTCTTATTGCTTCCACTATCCTATTCTTAAA
C_mouse AAAGCCAGAAATGAGGTCTAATGCCCCATCTCAGTTGACCAAGTCATTAGCGATGAGAATGTTTCTCTAATTTTTCAATATTTCTTCCTGGGCTGTGTACTCCCTTGCCCCTAAGGAATT
           * *   *        * * ****  ****    *****  ********   * * *   *** *    ***     ***   **      * *    ***  *  **       *
```

Zebrafish element D

```
                                                                                         K20
D_zebra CTTTCCCAAAACCAAAGTGAGCCTCAATAAGTGAGAAAAAAGAAAGGTCAGAGGAATTGCTGTAACTTCTTTACGCATTTGGAGGGGAAAAAATAAAAGGAGGAAATGACCCAATTTACA
D_zebra AATCCGCTCCAGGAATGGAATGTCTCCCAATACTGGTCAGGCATATGACAATACGACATAAAGCAAGCTGTCAAGTTACGCCCACAAAAATTTAGATGAGGGTGAATGGACACAAACCTG
                                         K20                                      K20                           K20
D_zebra CTCCAGAGCCAGCTGAGATCTGAAGACCAGCCGTGGGAGACTCCGCAACAACAGCAATTGTTCTTTTAACCGAGTAGGAGGCCGTTTTTCTTAGTGATTTTGTGTAGGTGTTACTTTTCT

D_zebra TTATAGATGCTCACAAAGCACTTTCCTGCATTTGTGCTATTGGTATTGGCAAGCAATTATTGTCCTATCTGCTTTTTTGGGGGAAATAACAAATCGGTTTGATTTTCCCATTTATGTACA

D_zebra AAAGGATTATTGAGACCGCATTCCATGGCCTGTAGAGAAATTCATTGTGTCCCTATTTTTAGCTTAATGCAAAATTAGTATTGAAGTTTTAACTCTTAAGTGATGTGAAGCCTATAATAT

D_zebra GATAATATTAAGGATTCTAACTTTCACTTTTTCTGCAGACAGATTTCTTATAGTTTTGGCTGGAACAAAAGTACACTGTAAAAAAACCTGGATGCCTTAAATTTTTAAGCTGAATCAAAT

D_zebra ATACCTTATGTGTCTATTGAACTTATATGATAAATGTTAACCTGACTTAAAACAGCTTGC
```

Alignment zebrafish-mouse core element E

```
                                                K20              K20                                                    K20
E_zebra ---------------GCATCTGAATGCTTCACATTTTCAGGGATTCTTGCGTCCTCCCCCACAAATGAAAGAAGGAAACTTCTTCA-GATGCATGTGTCTTTTGTTGTACGCCTACAGAT
E_mouse TTTCAAAATGTATGGACCTTCCCAAACAATTTCTATACACGGAGGCTTCAAACTTCGAATCCAGTTTCCCAAATGGTCTATATGGTTTAAAATGGGATTACGTGTTATTTCCCTATCGAC
                        * *    *  *      * * ** ***  ***    * **     **  *     ** *     * *     *     *  *    **** *   ****  **

E_zebra GCAAAAATGTTTGCACACTGTG--CAGAAGCGGCCCGCTTTGGCAGAGCGTGCCGGGCCCGCCGAATGCCCATTGGCAGCTGGGATCTGGCTGTCCCTGCTGGGAATGATGGTCTCAATG
E_mouse ACCAATATGGCCAATCACAATGTGCTAGCGGTAGCAAATTTATTCTTCTGTTGGGGGGTCTAGGGATCATCACATCCCTGCTGGGAAGGCTCAGTTCTCTCAGGCTTATGAACTCCCC--
         * ** ***      ***  **  *    *    *   ***        **   ***  *   * **   *     *     **    ***     **    **  *        *
                  K20
E_zebra TAATGGACTCCCACACATGGCAGCCACTGAATTCAAGAGCTTTCTTTCTTGCATGTGCTCGAGGTCTCGAGAGGTCAAATGACTTCTGCACATTGAGGATACATAGAATG
E_mouse ACCCAGCAACCCACACAACTCCAGGCTTCTAAAGAACAGTCCTTTGTCTCCACTCCGT---GAGTGTCAAGGAAGCAAAAGATTCAGCTGCACGG-CAATTAATCAAGTG
                 ********   *      *  *   ** **   * * ***    *  *      ** ** **    **** ** *      **      **  **  * **
```

Alignment zebrafish-mouse core element F

```
                                               Meis           HP Meis     Meis   Meis         HP
F_zebra GCAGCTCTGTTAAAGAGGAGGAAGTCAAATCTCCCAAACTGACATCAAGTCTTCATACATCCTCCATTCCAGCCCGACCTGGGATATGAATACAATGCAGGAAAATGCAAGGAATGCTTC
F_mouse --------------------GTGGGGTTGGGAGACAAAAGGCAATCAAGTGAGGATTGTCATTGCTGACATACTGACACCTGCATGTCAATGCCAAGGTGGGGGCTTCGAGGGCTCCCC
                            *  *          ****  *  *******    **      * *   *   *     *  * ** * *** * * *  **          *    *  *

F_zebra TCAATGTCATGGAGGGATCAGTGGCTCAGAGCGGCTGGCGGCTCCAGGAGCTCGGGATGGAAGGCCACCGGCGAGGGAAGCGAGCCCAGCAGACGTGAACCGCCAACTGGATGAGGTTCC
F_mouse TGGGATTCCCCACATGATCCCTCCATGTG-----TTTCTCCCATCACCCCACCATGGGGCAAGACTACCACGGAGGGTGGAGTGGCTGGCATACTGAACCTGCCATCCTGT-----GACA
        *     **       ****  *   *  *      *     *  **      *  *  * *** * ***   *****  * * * *  *** **   * * **** *  *        *
                                                                                       Meis                          Meis
F_zebra TCATACGACGGGCCCGCTTCAATCTTGATCCTCAACAATAACAGGGGGACAGGACAAGGCCATCAGTCTCCATTACGGAGACACTGTT-TTCTCTCAAATTACCCTCTCTGTCGTGTTCT
F_mouse TTTCCCTGGAGAAGACAGTTCAAATCCAGTCCCAACAATGCCCAGAAGACAGGGGAAGGCCATCAGTCTCCATTAGGGAGACAGGGTTATTCTCCCATATTATCACC-CTGTCATGTCCC
        *    *    *       *  *  *  *  * *******  *  *  ******  ******************** *******  *** ***** ** **** *  * ***** *** *
                               Meis    HP                                                                                Sp
F_zebra CCACAGCGTCT-ATAGGAGCGGCTGTCTCGTGCATCA-----------TTTCGCTCACAAAGCTTGAAAGCCCCCTGGAGGGGAGAGAGAGCTTCTTCCTGCCCTGTTGGCCCCTCCTC-
F_mouse CTTCAGTCTCCAAAAGGCATGGCTGTCTCATACATCATCATCAGCATGTTCCCCTCACAAAGATGGAGAACCCTACGGAGG---------GCTCCTTTCTGCCC---AAGTCCCTCCAGA
        *  ***  **  * ***   ********* * ******      **  ** * ********* * ** * ***   *****         *** *** ******       ******
                             Meis               Meis                                             Meis
F_zebra -----GCATGTCCGTCCACCTTGACAGGGACCGTCTCTGCTGACAAGGGGGGAAATTAGCATAGC----AATGGAGACCCTCTCAAGGCTGACAGGGGCGGACATTGTACTGTAAG
F_mouse GATAGCCAAGGGCCTTCCCCTTGTCTGAAAGGATGCTAGACTGCCAACAGAGACATCTGCATCCTAATTAAAGGCTAGGCTTTGAAGAGTGCCAAGGAGGGACCTGACCTCCAGTT
              ** *  * * * ***** * *  *   *    *    * *   * ** **  ****       ** **  *  ** * ***  ** ** **  **** *
```

Alignment zebrafish-carp koi element A

```
                    HP          HP
A_zebra     ATTATATACATCATTTTTGATACAT-----TTTAAAATGTGTGTAGCATGTACAAAACATATTTCATTCTTCCACTT-------ACCACTAGAAGGAAGCCTGCGTACCACAGTTTGAGA
A_carp      ATTCACACCAATTGCTTTAATGTACTGTACTTTAGAGAATGCATGGTG------ATATCTATTAGTTTTACACTTTGTATTCACCTGTAGTGTTTTGCTTTATCACTGGTGTTTTTCA
            ***     **     *** **  *      **** *  *  ** *   **      * ** * * * * ** *****       ***  ***      ** *    **    ****   *
                                                                                         Meis
A_zebra     ACCACTGCTCTAGAGTAATTTTCAAGACACGATTCCAATCCACTCCAAACCAGTTTCTGTGTGAAAGCCTCACACCTGACAGCGGTGAGATCTGTCTGCTAGTTTAACATGTGTAAGAAT
A_carp      AGGCTTGA-------TTCTCATCCACTGTATATCCAGTTTGAACCACTTGAGAAAGTTGTGAGAAAGCTGAACACCTGACAGCGGTGAGAGCTGTCTGCTAGTTTAACATGTGTAAGAAT
            *    **        *  *  ** *      ** *   *  *  *            **** ******   ******************* *****************************
                Meis                                       K20                K20                                     K20       K20
A_zebra     ATTTGTCAGGCACATTAGCTCACCGCAGGGTTCCCATTGTGTCCCCCGAGGAGGCCTTAAAAAATCCCCCCTCACATCCCCGTTGGCTCTTTTAGGGCGCTGTGTGTAGGAGGATATGTG
A_carp      ATTTGTCAGGCACATTAGCTCACTGTGGGGTTTCCATTGTGTGTAAGGGGGCGGCCTTCA--AAACCCCCATCACATTCCCTTTCTGTCTTCTAGGGCGCTCTGTGTAGGAGGCTATGTG
            *********************** *  ***** *********     * ** ****** *  ** ***** ****** *** **   **** ********* *********** ******
                                         K20                                      Mafb
A_zebra     GGATTCCCCATACAAGCGCTCCAAAAGCACACAGGAGAACCTG----------GACAGAAGACCCGCTGTAAGCAGAATGTCTGGAGTCGGGACTCTCCTCTATAG--------------
A_carp      GGATTCCCAATACAACAGCTCCAAAAGCACATAGGAACCTGCATCTGCTCGTCGACAGAATACCCGCTCTAAGCAGAAACCTTCGTGTCGGGACAGAGGAGAGCACAGAGGACAAAGAAA
            ******** ******  ************** ****                 ******* ******* *********    * * ********          *
                                                        K20               K20           K20                     Mafb
A_zebra     ------------------------------------------------------------------------------------------CTTGGCTGGCTCATCATTACTTTTGAGGGC
A_carp      GCGGCCGAGGAGCAGCAGTTATTCAGTCAGACTCGGGAAGAGTGTGGGCTGCAGCATTCTGCCTCCTGAGTAGCTGGGGGTCTTCTATGGCTTGGCCGGCTCATCATTACTTTTCAGGCC
                                                                                                      ****** ***************** *** *
                                                    K20                                                   Mafb    Meis
A_zebra     CGAATCGAGGCAGGTGGAAGAGGCGACGGCTCGTCCTCCTACACGCAGATTCCAGTGGACGTCAAGCTCTCGTTTTCTCACAGCTGTCGACTGCTGATGTGCTGACAGAAGAAGAGCTGG
A_carp      AGACTTCAGCCAGGTGAAAGAGGTGACGGGCCATCCTCCTACACGCAGATTCCAGTGGACACTAGCTGTT-----------------------GTGACGTGCTGACAGAAGAAGGGCGAC
             ** *  ** ****** ****** *****  * ***************************   *     *                        *** **************** **
                                K20           Mafb    Mafb
A_zebra     AGAT-----GAACACACACACACACACAGGAGAGAAGAATAGCTTAGTACTGACTTGATGCTGTGCGCTCAAATGATTTAGCTGCTTGTCGAAATTACTTATTTAAAATGAGCTGAAAC
A_carp      AGGAAGAGCAGGGACTGCCACACACATAATAGAGATTACTAGCTTACTACTTGCTCAATACTGTACAGTATGCACTCTACA----------------------------TCGCCTGTGGT
            **           **    ******** *  *****  * ******* ****  **  ** **** *  *        *  *                            *   ***
                         K20                                              K20                  vHnf1
A_zebra     AACACAATTCTGGAGGTGTTTTTGGTGGAGACCTAATTGTTTTACTCTGCATTTTTATACTGTGTGCTGCACTCTAACATAATCATTAAGTAAGTTAACTATTTTTTTCCAATTTAAATG
A_carp      AACACATTATT--------------CCCCACTATAGATATTATACTA----------ACATGTCACCTGCCATGCCAGTCATTAAAGTATTAG-CTTAATTTCTGCTTAAACTTTATTTT
            ****** *  *                      **  * **  ****              ***   ****  *   *   * * *   *  **   * * * * *  **  * ****  *
                                                                                     K20                                      Mafb
A_zebra     G--------------------------------------ATTAAACTTTTTAAAAAAATAAGTTGTCCCCCGCCCCCAACACCCCCCCCCCTCTCTCAAAAAAAAACAAGAAATGTGTTG
A_carp      AATGGTCCTCCTACAAACTATAAGTAAACTATAAACTATATGTAACTTTGTAAGAACGTATCAACTTGTACTAACCCTAACTGTAACCCTAACTGTAACCCTAAAGAGAGTTAGCTGACA
                                                   **  ****** *** **  **     *    *   *** *       ***   ** * *    ***   **  *  **
```

S1 Fig. Sequence alignments of enhancers between different species and search for putative transcription factor binding sites.

The first panels show sequence alignments of zebrafish and mouse entire element A and core regions from elements B, C, E and F. The non-conserved element D sequence is also shown. The last panel shows a sequence alignment of zebrafish and koi carp element A. Conserved nucleotides are marked by a star, and characterized or putative binding sites for transcription factors are indicated: Krox20 (K20) in red, Mafb in blue, vHnf1 in green, Hox/Pbx (HP) in purple, Meis in pink, and Sp in orange.
