# Supplementary material for: Cooperation, cis-interactions, versatility and evolutionary plasticity of multiple cis-acting elements underlie krox20 hindbrain regulation
Source: PLoS Genet. 2018 Aug 6;14(8):e1007581. doi: 10.1371/journal.pgen.1007581 (PMC6095606; doi:10.1371/journal.pgen.1007581)
Supplement: S2 Fig — Each panel shows the entire (A to F and krox20) or partial, (D-E) and (A-E), nucleotide sequence of the considered region. The DNA fragments with enhancer activity that were used to drive GFP expression in transgenic lines are shown in green. The target sequences for the guide RNAs used to generate the germline deletions are shown in red and the sequences eliminated in those deletions are indicated by capital letters. The target sequences for the guide RNAs used to generate the somatic deletions are shown in blue. Putative Krox20 binding sites in elements A, D and E are underlined (pink). The sequence encoding Krox20 zinc fingers is underlined (black) and the single nucleotide mutated (G to A) in krox20fh227/fh227 is shown in orange. Diagrams above the sequences indicate the approximate positions of the targeted sequences within the locus. (PDF) [file pgen.1007581.s002.pdf]

A

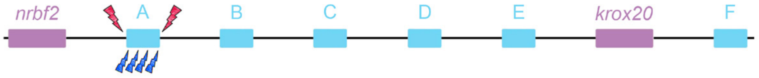

aaaatcctttcttttgagtgtgtacaagtaaataatgagcaaataattgcagaaatgtgtgtgaactatcccttTAATGCTCTAGAGCAGTGGCTCTCAAACTTTTT
TCATCAAGTACCACCTCATAAAAAAATTGTCTCTCCACGTACCACCAAAATGAGCAGTATTGAAATACAATAGCGTAGTAGGCCCAGTAAAGCAGCTACAACTCTGC
ACAGTTTAAAAAACGTGGCAAATTACCTCAGAAATATAGGCTATATGTCATATATGGCATATTATATACATCATTTTTGATACATTTTAAAATGTGTGTAGCATGTA
CAAAACATATTTCATTCTTCCACTTACCACTAGAAGGAAGCCTGCGTACCACAGTTTGAGAACCACTGCTCTAGAGTAATTTTCAAGACACGATTCCAATCCACTCC
AAACCAGTTTCTGTGTGAAAGCCTCACACCTGACAGCGGTGAGATCTGTCTGCTAGTTTAACATGTGTAAGAATATTTGTCAGGCACATTAGCTCACCGCAGGGTTC
CCATTGTGTCCCCCGAGGAGGCCTTAAAAAATCCCCCCTCACATCCCCGTTGGCTCTTTTAGGGCGCTGTGTGTAGGAGGATATGTGGGATTCCCCATACAAGCGCT
CCAAAAGCACACAGGAGAACCTGGACAGAAGACCCGCTGTAAGCAGAATGTCTGGAGTCGGGACTCTCCTCTATAGCTTGGCTGGCTCATCATTACTTTTGAGGGCC
GAATCGAGGCAGGTGGAAGAGGCGACGGCTCGTCCTCCTACACGCAGATTCCAGTGGACGTCAAGCTCTCGTTTTCTCACAGCTGTCGACTGCTGATGTGCTGACAG
AAGAAGAGCTGGAGATGAACACACACACACACACAGGAGAGAAGAATAGCTTAGTACTGACTTGATGCTGTGCGCTCAAATGATTTTAGCTGCTTGTCGAAATTACT
TATTTAAAATGAGCTGAAACAACACAATTCTGGAGGTGTTTTTGGTGGAGACCTAATTGTTTTACTCTGCATTTTTATACTGTGTGCTGCACTCTAACATAATCATT
AAGTAAGTTAACTATTTTTTTCCAATTTAAATGGATTAAACTTTTTAAAAAAATAAGTTGTCCCCCGCCCCCAACACCCCCCCCCCTCTCTCAAAAAAAAACAAGAA
ATGTGTTGTTTCCAACATAGGCTCTTTAAAGAGTGTACTATCTCAGTATACCGTCTTCTTGTCAGAGAAAATAAATATAAAAATAAATTGTAGTAATGTGTGAAGGT
ATTGGTCACCTACACTGTAAAAATGCAGGGTTCCACACAATTCCCAACACAAACTGATTAAGTTAACTTAAAACTTTTAACAAATTTATGTGGATTAAACAGAAAAA
AATAAGTTGTCCCAATTAAATCTCAAGAATTCTGTTGTTTCAGCTCATTTTAAAGAAATAGTTTGAATGAGCAAAAAAAAAAGTTTTTTTTGAGTGGAACAGTTTTT
GTTGggtacatttaaaaaaatatatattcttacggccacaactgcattgtggatacatcggtgccgctttagaggcgttgctcgctgtagaagtgagaa

B

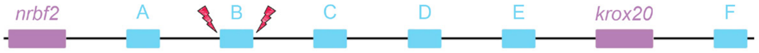

aatatactaatcatataatataatataatataatataatataatataatataatataatataatataatataatataatataatataatataaggtaaaatctcccaccatc
gacccacacctaaaagcgcaccccgtcagtcccatcactgtcttttgttcatttctccaggcatcagcaggacagagtactgtatatccttgtcctGTAAACTCCCC
CTTTATCCCACAACATCCTGTGACTCCCTCATTCCCCCAGTCCATGAATGTCCCCAATACAGTCCAAAACTACAGACAGGGCAATTTTCTCTGACGTGCTCTGCCAT
CTTGTTTTGCACAGCGGGACAAAGGAGGTCCAAATGATGCCTTTGTTGGAGCAGACGAACCCTTGACAGACGAGCTCCAGATCCAAAGCCTGCTCTTCCTGCTCTGC
TTTGGCCGGTGGGTTAATCTTCAACCAGGTACAGGAACTGACCGTTGTGCTTACGTAGTGGAAGTGTGTGTCCATCCGTCTCCCCTGACAGAAAGCTCCACTTAGCT
TGTCCTGGGCTTGTAACGCGCCATGTCCAATTAGCTCTCGGGGTGCTCCGTCTTCATCCCTAAAGACTGGAGAAAGCGCGTGGACCATGATGGAGGAGGCCTTGATG
GGACAACAAGACGTCCTGCAGCCGCAGTCCAGACTGAGTCGGCTAATCATCTACTGCATGCTCAGCTCTGGAGGTGAAAGTCTGTGGCCCAGAGAGGAAGTGTCAAA
CTGGGCCCTCAGTGCCAGGCCGAGTGGGACAGAGACGCCGCTATTGTTTTTCGGCGGTGCATTTCTGCTGATGAGGACTTTGGCTGTGTTCAGCAGGAAGTGGAGAT
GTGGAGGTGTTAGTGGTTGTCAGAGTTCATGAAGAAGGTTTGACCACACAGCTAAAGTTTTATTACTTCAGTTTTACAGAAACTCTACAAGACTTGAGTTTTACTTG
ACAAGACTTTCTGTTATAACTAAAGAAATACACAGTAAAAATGCTTCGTTGTTTTGATGGACCACTTCAAACATTGTTAggtctgtctgtctgtctgtctgtctgtc
tttttggtaacactttacaataaggttcattagttaatgcgtttactaacat

B’

aatatactaatcatataatataatataatataatataatataatataatataatataatataatataatataatataatataatataatataaggtaaaatctcccaccatc
gacccacacctaaaagcgcaccccgtcagtcccatcactgtcttttgttcatttctccaggcatcagcaggacagagtactgtatatccttgtcctgtaaactcccc
ctttatcccacaacatcctgtgactccctcattcccccagtccatgaatgtccccaatacagtccaaaactACAGACAGGGCAATTTTCTCTGACGTGCTCTGCCAT
CTTGTTTTGCACAGCGGGACAAAGGAGGTCCAAATGATGCCTTTGTTGGAGCAGACGAACCCTTGACAGACGAGCTCCAGATCCAAAGCCTGCTCTTCCTGCTCTGC
TTTGGCCGGTGGGTTAATCTTCAACCAGGTACAGGAACTGACCGTTGTGCTTACGTAGTGGAAGTGTGTGTCCATCCGTCTCCCCTGACAGAAAGCTCCACTTAGCT
TGTCCTGGGCTTGTAACGCGCCATGTCCAATTAgctctcggggtgctccgtcttcatccctaaagactggagaaagcgcgtggaccatgatggaggaggccttgatg
ggacaacaagacgtcctgcagccgcagtccagactgagtcggctaatcatctactgcatgctcagctctggaggtgaaagtctgtggcccagagaggaagtgtcaaa
ctgggccctcagtgccaggccgagtgggacagagacgccgctattgtttttcggcggtgcatttctgctgatgaggactttggctgtgttcagcaggaagtggagat
gtggaggtgttagtggttgtcagagttcatgaagaaggtttgaccacacagctaaagttttattacttcagttttacagaaactctacaagacttgagttttacttg
acaagactttctgttataactaaagaaatacacagtaaaaatgcttcgttgttttgatggaccacttcaaacattgttaggtctgtctgtctgtctgtctgtctgtc
tttttggtaacactttacaataaggttcattagttaatgcgtttactaacat

C

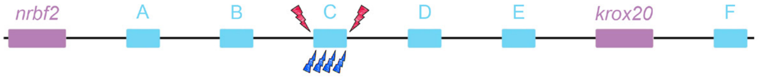

tactaattctgctataatgaagttatttgcatccagcagaatgcctaaactcaatacctatgtacctatgatccagtctctgtgctctctgggataaaggtaaagtc
agagctgcagagctgttgtccAGGGGTGAAGACACAAAGGCCCAGTGGCAGGTCTTTTCTCCCCCAAAGCCGATACCACCTGCAGGGCCCGGGCCTCTCCTGAGTGT
CCCAGCACCACTCTCCAAATGATGATTAATCACGGAGTGCTAAGAGGGGGGGGGTGGTTGCACCCTGACACAGCCATAATGCTGGTCACCAGAGTCTGGATTGGCCA
GCGGTGGTCAGTGGGTGGATGTCCGAGGAAGATGGATGACCTCGACCTCCTGTCATTAACAAGAGAAGAAACAAAACAGACAACAAAAAGAAAGGCAGCACGGTGTG
AGGGAGAGTTTGTGTGGGAATAAATGAGAGTTTTGACATTAGCGAACGTAAAGTAAACGCAAAGTAGTCCCTGACACGACATGACAGCGCTGAACTTGTGGAAGTTT
GAGGATCGTTTGCTCTCATCAGCGGAAAGAGGAGCCGAGGTTTCTCTGACAGTTACAAATGAAGCCGCGATTCATTATTTTCCAGGGAATTCGCTCCTGCGCAGACG
AAGATGGAGATGCAGCCGTGGCGTCACTGCCCATTTGGATGGAGATGAATCAAGGCGCTTTAGCCCAGACGCAGCACTGACAATCACATCAGCGAGGCTATCGGACT
CCCCTCTACTCCTGGTGGACGGATCCGGTGCGGAGCGGAGCGGGTAATAAAGGCTCCTACTGAAACCCCATTAAGCCCGGTCTCCAAAGACCAGCTCATTAGCATAG
CGGAATATTCCCCCCGTTTGTGTAATTCTATCAACTCTTATTGCTTCCACTATCCTATTCTTAAATGACGCTAATAATGTGGAAAGTTTAGCTGTATAAATGAGGCT
GTTCTTGCGCTTTTGCAGCTTGTGTTCATTTTAATCGGGGGTCTGGTTACCTCATTCACAATCCCCCTAATGACCCATCTTTGTTAATTAAGCAGACGAGGACTGGT
GTGTGTGTGTGGGGGGGGGGGGGTGCACATGCAAGAAAAGCCACAATTCTCCTTTGTTGTCAAATTGACACCAGTGTCTGTTGTCTGTCTGAAATTAAGGACAGATT
TGGATACAAGCATGCTATTTGGCATTTAGCACATGTAATAGACGTGAAGCTGTAAATTAGGAACAAATCAGTGACTTTTAAATCAGTTATTTCATGATCACTACCAT
TGAAAAATCAGGGAAAAATGTAATTATTCCACCATCTACCTattcttttattcccacatccaataatgtttgagaatcatgtagtttaagtgttcttgctgcc

D

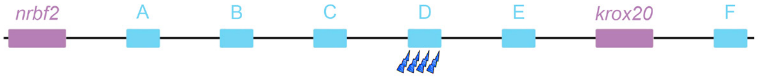

ctatctatctatctatctatctatctatctatctatctatctatctatctatctaatcaagtcattttatcatggaatctattttctttcccaaaaccaaagtgagc
ctcaataagtgagaaaaaagaaaggtcagaggaattgctgtaacttctttacgcatttggagggggaaaaaataaaaggaggaaatgacccaatttacaaatccgctc
caggaatggaatgtctcccaatactggtcaggcatatgacaatacgacataaagcaagctgtcaagttacgcCCACAAAAAtttagatgagggtgaatggacacaaa
cctgctccagagccagctgagatctgaagaccagccgtggGAGACTCCGCAACAACAGCAATTGTTCTTTTAACCGAGTAGGAGGCCGTTTTTCTTAGTGATTTTGT
GTAGGTGTTACTTTTCTATAGATGCTCACCTTCAAAGCACTTTTGCATTTGTGCTATTGGTATTGGCAAGCAATTATTGTCCTATCTGCTTTTTTGGGGGAAATAAC
AAATCGGTTTGATTTTCCCATTTATGTACAAAAGGATTATTGAGACCGCATTCCATGGCCTGTAGAGAAATTCATTGTGTCCCTATTTTTAGCTTAATGCAAAATTA
GTATTGAAGTTTTAACTCTTAAGTGATGTGAAGCAAGGAAACTTTCACAGTATTTCCTATAATATATATTTTTTTCTGCAGACAGATTTCTTATAGTTTTGGCTGGA
ACAAAAGTACACTGTAAAAAAACCTGGATGCCTTAAATTTTTAAGCTGAATCAAATATACCTTATGTGTCTATTGAACTTATATGATAAATGTTAACCTGACTTAAA
ACAGCTTGCATAATTTATAAAAttaagttataacatgataaacttagtttaatatgttaataaactaaaaacatatgttgtcaggactaattgatcatacttttttt
tttacagtgtactttaactataaaaaatctgtctccagaaggaaaatgtattagtattattgttagtgcaatttaaagtctcaactaattaggttaattaggcaagt
tactaataatattgaccataaaattattacaaaaaatgttattccagctaaaactagaagtaataaaactgtttccagaaggaaaaaatattagtattattatttat
aattattagctttccagtgattaaaaaaatatttttcccaagtgcttttgtaaatggattgttttaaatagttttaataactagttttaaat

E

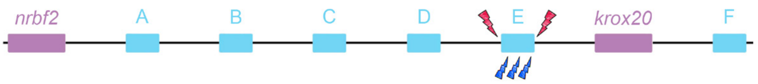

gccctgcaggcacgtttttacgcagcggatgcccttccagccgcaacccatctctgggaaataattcagattctatatgtttttttaaaaaagttatgttgatttat
tttttttgtttctgtttcttacatttcataaatccccaaagaccacacttgcagctaaaaggttcaactactgaagtagaaattcacCTATACTCTTGGTTGAGGGT
GAGTAAACTAACAACTATTTATTTTTGGTGAACTACGCCTTTAAAGGGGATAACTGTCTCATTGACATCCGAAGTTTCTGATAGTGTACATAATGCATTATGGATCT
TTCATATTCATAGCTGCACTAATAAGATCACAGCTGCTATAATATGAACTTTACTATTACTAAGTGTTCAATTGTCACTGTAAGACAGCTCCAGAAGAAAGCTCAGC
TCGACACACAAAAGCTCTCAATTTTGCACAGAACAGCGACAATCTTTGCCACTGACTGGCGCATCTGAATGCTTCACATTTTCAGGGATTCTTGCGTCCTCCCCCAC
AAATGAAAGAAGGAAACTTCTTCAGATGCATGTGTCTTTTGTTGTACGCCTACAGATGCAAAAATGTTTGCACACTGTGCAGAAGCGGCCCGCTTTGGCAGAGCGTG
CCGGGCCCGCCGAATGCCCATTGGCAGCTGGGATCTGGCTGTCCCTGCTGGGAATGATGGTCTCAATGTAATGGACTCCCACACATGGCAGCCACTGAATTCAAGAG
CTTTCTTTCTTGCATGTGCTCGAGGTCTCGAGAGGTCAAATGACTTCTGCACATTGAGGATACATAGAATGACATCCTTCTGCATCAGCCTTTGCATTGTTTTTCCT
CTCTGTTTCATTGTTCTCTATTACTCATCAAGTTGGCTGTGAGATCGGCACACACAGCAAAAGGCAAAATCAGACAACTGTACTCACAAATTAAGTAAATAGGTCAG
TTTACAACCTTTATCTTTATTACAGGTTACTTAATTTGTCAAATAATTTCTAGGCACATAATTTGCGAAATTTAGGCTGTGTACACTGTAAAAAAAAAGACAATCAA
CATTGTCTTATTCATGGATTACATTACTCAAAAAAAAAAAAAAAAAACTACATAGAATCTAACAAAATATCTCAGCTCTTTGGGTCAAATGTAATTCACTAGCTGCT
GATCTACCTCAATTTTTTGAATTGGGATTTTTGAAGTGCTTTCTGAAATGTTTAAGCTGCAATTACTGCCAAACCCAATGCTTATAATTCTGGGATTTTCTGAAGCA
TTTGTTGTCAAACACACAAAAcaaactgctctcatgcagcttaactatggagccagatcactctcaagtcgacgc

F

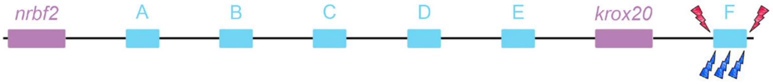

tgctagctgggctatttcacacaattttgatttcatactgaatttaaacctcaggatatctttaggaagcattcggctttattcatttacttgcatatcactaaaga
caaCGGCTTCTGCTAAAAAATCACCTTTAATATCCTACTAAAGCAATAAAATTACTACATCTTTAATGACCTGAGGAGGACTAAAAAAACAGCTTTTGGGGTGAAC
TATCCCTCTAATGCGTCTCTTTGCGAAAGACAGGAAATGATTCATCACTCCAACCTTTTCTTCATGAGGCATTCGTACCACTATTATCATTAGTGTTTCATGCATAG
TAAAACCTCAGCGGTTTGATTAGAGTCACGAAGCATGGCCTGTTACATTGTCAGCTCCCTGGTAAACATCCATTATTCTGTTCCACAGACTCACACGCACACACATA
CAATCAGCAGCTCTGTTAAAGAGGAGGAAGTCAAATCTCCCAAACTGACATCAAGTCTTCATACATCCTCCATTCCAGCCCGACCTGGGATATGAATACAATGCAGG
AAAATGCAAGGAATGCTTCTCAATGTCATGGAGGGATCAGTGGCTCAGAGCGGCTGGCGGCTCCAGGAGCTCGGGATGGAAGGCCACCGGCGAGGGAAGCGAGCCCA
GCAGACGTGAACCGCCAACTGGATGAGGTTCCTCATACGACGGGCCCGCTTCAATCTTGATCCTCAACAATAACAGGGGGACAGGACAAGGCCATCAGTCTCCATTA
CGGAGACACTGTTTTCTCTCAAATTACCCTCTCTGTCGTGTTCTCCACAGCGTCTATAGGAGCGGCTGTCTCGTGCATCATTTCGCTCACAAAGCTTGAAAGCCCCC
TGGAGGGGAGAGAGAGCTTCTTCCTGCCCTGTTGGCCCCTCCTCGCATGTCCGTCCACCTTGACAGGGACCGTCTCTGCTGACAAGGGGGGAAATTAGCATAGCAAT
GGAGACCCTCTCAAGGCTGACAGGGGCGGACATTGTACTGTAAGTGACTCTACCGTCGTACTGTGGTCCCCCCCACCCCACAAAACCTACCATGACAAGGCTCTCTA
AAAGTTGTTGAATCATGAAAACATGTGACCATATATCATAAAAATTATGTCTTATGTTGCAGAGGTTTATTTTTTTGGCAGTTACCAAAAAACACTATCAATGTTAA
AGATTTTTCTTTTGTGGCAAAATTCATTTGAATATTAAGTAAAGATTGTGTTTCGATGAAAATTTTTAGTAAATTTACAAACGTAAATACATAAAAATGTAACTTTT
GATTACTTATATCCATCTCTAAGCACTTAATTTAGACAatttaaagacgattttctaggaatttagatttttttaaccctcagagtacagaatttcaaatatttg
tatcttggccacatattgtcattgtcatatcctaacaagggatttatgacaaaacacttactgtatcactggttggt

(D-E)

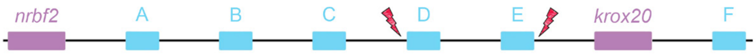

ctttattaatctggggtcgccacagcggaatgaaccgccattccagcacgtttttacgcagcggatgcccttccagccgcaacccatctctgggaaataattcagat
tctatatgtttttttaaaaaagttatgttgatttattttttttgtttctgtttcttacatttcataaatCCCCAAAGACCACACTTGCAGCTAAAAGGTTCAACTAC
TGAAGTAGAAATTCACCTATACTCTTGGTTGAGGGTGAGTAAACTAACAACTATTTATTTTTGGTGAACTACGCCTTTA ••• GAACAAAAGTACACTGTAAAAAA
ACCTGGATGCCTTAAATTTTTAAGCTGAATCAAATATACCTTATGTGTCTATTGAACTTATATGATAAATGTTAACCTGACTTAAAACAGCTTGCATAATTTATAAA
ATTAAGTTATAACATGATAAACTTAGTTTAATATGTTAATAAACTAAAAACATATGTTGTCAGGACTAAT ••• AAATATTAGTATTATTATTTATAATTATTAGC
TTTCCAGTGATTTAAAAAAATATTTTTCCCAAGTGCTTTTGTAAATggattgttttaaatagttttaataactagttttaaatgtattaaaataaattagtcatgtt
ctaacttcattgtataagtttcgctagctgttttaa

(A-E)

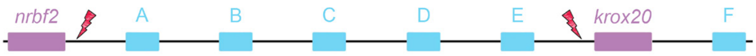

cccctggtctagactgagcataatcctcatttttttgaagagtatctcattatcagttcaataaattgagcatttttgtgttcttcaaccatgacagaagcttgaatg
ggagtctaaatcggccccacacacaggaaacctcagatcacgccccccctgttttgatagtacaataaaaaaaaatccccataGCTTCCTGTTGTCCGTCAATCAATG
AGTATTTTTATCGCCACCCATGCAAGCTCGGGCTATTTTTGAGAACGTGTTGCTGTC ••• ATAAGTGCACTCTCTGAATGGACAAAACATTACGATGAGCAAGAA
ATCTCAAACCACTGGATAGAAAACCCTAATGTACATTGTcaaaaatgcacacagtgtttggacagcatgaagaacttaagttgacttgttagtttttacacatttaa
gtgaatttaacataaaacagttaagatatatacacaaggtgggccatttgtatggatactccttaataaaataggaatggttggggattttaacgtcctgtttgtgg
cacattagtatatgtgagggggcaaacttttcaagatgggtgatgaccatggtgg

krox20

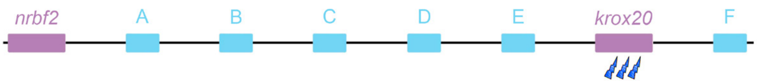

agttgaaagttcactggtttgaactggacgagcagatgctcgtggcgacgccgcgctctgagctggacactcctgtggacgacgaggaggactttcgctctttttgt
cgcaggtggatttggtgtgtctctttctttcgtcgctcctcgcgaactttctcccgcaaaagtcgcaggcgaacggcttttcgccggtgtgcgtgcgaatgtgggt
cgtcaggtggtcgctgcgactaaagttcctcatgcatatccgacactggaagggtttgtggccggtgtggatgcggatgtgtctggtaagttcgtccgagcgcgaga
agcgcctgtcgcagccctccgccggacaggggtagggccgctcgtgcacgggcgtcttgctgggtctgttggggtatttccttggacgcaggattggcctgaggggc
aaattctgcggagtgtaagctgtgggtagccggggtccgtcaggcactggtccccctagcgtgaagttcctgatagtgttcaggggggttagaggaggcggtaattt
gaaagagtccagcgggcaggagaacggtttgcggtcgggcatcgactgcatgtccctctggcacggaggttggaaaaagccggcgtagtccgggattatagggaaca
acccagagtcggcgtttggctttggagaggagtaggatggcggaggatatgagatgggacaggtggaggtggaaaggaacgcagacgggtcttgatagacctctccg
catccagagtagggcggcgggggggagtagatgtgctccatgtctgcttggttctgcgccatggtgcagctgagagtgctggagaagtggctgggggagactgagga
tgcaggtgacgaggatgctgaggatggctgggtcatgcccaggatccccgcgctcacgatgttgatcacgccctctgggttccagtttcccgggtactgggagtcga
tggaaaactttcccatgtaggtaaaagtttggttgcgagggccagctggttgcgcgaagctgctggagtaggctaagtcgagggcgcgcttctccgtgctcatatcc
ccgttaatcaggccat

S2 Fig. Sequences of the different regions of the zebrafish krox20 locus in which deletions were introduced.
Each panel shows the entire (A to F and krox20) or partial, (D-E) and (A-E), nucleotide sequence of the considered region. The DNA fragments with enhancer activity that were used to drive GFP expression in transgenic lines are shown in green. The target sequences for the guide RNAs used to generate the germline deletions are shown in red and the sequences eliminated in those deletions are indicated by capital letters. The target sequences for the guide RNAs used to generate the somatic deletions are shown in blue. Putative Krox20 binding sites in elements A, D and E are underlined (pink). The sequence encoding Krox20 zinc fingers is underlined (black) and the single nucleotide mutated (G to A) in krox20fh227/fh227 is shown in orange. Diagrams above the sequences indicate the approximate positions of the targeted sequences within the locus.
