# Supplementary material for: Cooperation, cis-interactions, versatility and evolutionary plasticity of multiple cis-acting elements underlie krox20 hindbrain regulation
Source: PLoS Genet. 2018 Aug 6;14(8):e1007581. doi: 10.1371/journal.pgen.1007581 (PMC6095606; doi:10.1371/journal.pgen.1007581)
Supplement: S3 Fig — (A) PCR amplification of either wild type (WT) embryos or F0 injected embryos (MUT), using the primers indicated in S1 Table. F0 embryos were injected at the one-cell stage with CRISPR/Cas9 ribonucleic complexes targeting elements A, C, D, E, F or the krox20 gene and PCR amplifications were performed on whole embryos at stages 12s (for element A, and the krox20 gene) or at 22s (for elements C, D, E and F), corresponding to stages of in situ hybridization analysis. Complete elimination of the wild type diagnostic fragments demonstrates the high efficiency of the procedure. (B) Sequence alignments of wild type element C (WT) with five mutated alleles generated by CRISPR/Cas9 injections. Red brackets indicate the limits of the deletions and putative binding sites for transcription factors are indicated: Hox/Pbx (HP) in purple, Meis in pink, and Sp in orange. (PDF) [file pgen.1007581.s003.pdf]

A

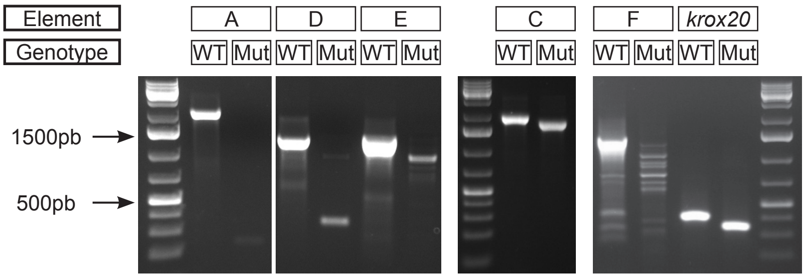

B

```
WT      GGGATAAAGGTAAAGTCAGAGCTGCAGAGCTGTTGTCCAGGGGTGAAGACACAAAGGCCCAGTGGCAGGTCTTTTCTCCCCCAAAGCCGATACCACCTGCAGGGCCCGGGCCTCTCCTGA
Del 1   GGGATAAAGGTAAAGTCAGAGCTGCAGAGCTGTTGTCCAGGGGTGAAGACACAAAGGCCCAGTGGCAGGTCTTTTCTCCCCCAAAGCCGATACCACCTGCAGGGCCCGGGCCTCTCCTGA

                                            HP                                         Meis
WT      GTGTCCCAGCACCACTCTCCAAATGATGATTAATCACGGAGTGCTAAGAGGGGGGGGGTGGTTGCACCCTGACACAGCCATAATGCTGGTCACCAGAGTCTGGATTGGCCAGCGGTGGTC
Del 1   GTGTCCCAGCACCACTCTCCAAT---------------------------------------------------------------------CCAGAGTCTGGATTGGCCAGCGGTGGTC

           Sp               HP
WT      AGTGGGTGGATGTCCGAGGAAGATGGATGACCTCGACCTCCTGTCATTAACAAGAGAAGAAACAAAACAGACAACAAAAAGAAAGGCAGCACGGTGTGAGGGAGAGTTTGTGTGGGAATA
Del 1   --------------------AGATGGATGACCTCGACCTCCTGTCATTAACAAGAGAAGAAACAAAACAGACAACAAAAAGAAAGGCAGCACGGTGTGAGGGAGAGTTTGTGTGGGAATA
```

```
WT      GGGATAAAGGTAAAGTCAGAGCTGCAGAGCTGTTGTCCAGGGGTGAAGACACAAAGGCCCAGTGGCAGGTCTTTTCTCCCCCAAAGCCGATACCACCTGCAGGGCCCGGGCCTCTCCTGA
Del 2   GGGATAAAGGTAAAGTCAGAGCTGCAGAGCTGTTGTCCAGGGGTGAAGACACAAAGGCCCAGTGGCAGGTCTTTTCTCCCCCAAAGCCGATACCACCTGCAGGGCCCGGGCCTCTCCTGA

                                            HP                                         Meis
WT      GTGTCCCAGCACCACTCTCCAAATGATGATTAATCACGGAGTGCTAAGAGGGGGGGGGTGGTTGCACCCTGACACAGCCATAATGCTGGTCACCAGAGTCTGGATTGGCCAGCGGTGGTC
Del 2   GTGTCCCAGCACCACTCTCCAAATGATGATTAATCATGCGTCAATGGGCGACGATGATTGACGCAAATGATGA----------------TTA--------ATCATGCGTCAATGGGCGAC

           Sp               HP
WT      AGTGGGTGGATGTCCGAGGAA------GATGGATGACCTCGACCTCCTGTCATTAACAAGAGAAGAAACAAAACAGACAACAAAAAGAAAGGCAGCACGGTGTGAGGGAGAGTTTGTGTG
Del 2   GCAGGGTGGATGTCCGAGGATGATGGATGACCTCGACCTCGACCTCCTGTCATTAACAAGAGAAGAAACAAAACAGACAACAAAAAGAAAGGCAGCACGGTGTGAGGGAGAGTTTGTGTG
```

```
WT      GGGATAAAGGTAAAGTCAGAGCTGCAGAGCTGTTGTCCAGGGGTGAAGACACAAAGGCCCAGTGGCAGGTCTTTTCTCCCCCAAAGCCGATACCACCTGCAGGGCCCGGGCCTCTCCTGA
Del 3   GGGATAAAGGTAAAGTCAGAGCTGCAGAGCTGTTGTCCAGGGGTGAAGACACAAAGGCCCAGTGGCAGGTCTTTTCTCCCCCAAAGCCGATACCACCTGCAGGGCCCGGGCCTCTCCTGA

                                            HP                                         Meis
WT      GTGTCCCAGCACCACTCTCCAAATGATGATTAATCACGGAGTGCTAAGAGGGGGGGGGTGGTTGCACCCTGACACAGCCATAATGCTGGTCACCAGAGTCTGGATTGGCCAGCGGTGGTC
Del 3   GTGTCCCAGCACCACTCTCCAAAGACCAACGGCCACTGACTTACTGGTGCCCAAC---------------------------------------AT----CCCGGGGTAAGGGGTGGAAC

           Sp               HP
WT      AGTGGGTGGATGTCCGAGGAAGATGGATGACCTCGACCTCCTGTCATTAACAAGAGAAGAAACAAAACAGACAACAAAAAGAAAGGCAGCACGGTGTGAGGGAGAGTTTGTGTGGGAATA
Del 3   ATTACATTGACGCAGGTTGCAGATGGATGACCTCGACCTCCTGTCATTAACAAGAGAAGAAACAAAACAGACAACAAAAAGAAAGGCAGCACGGTGTGAGGGAGAGTTTGTGTGGGAATA
```

```
WT      GGGATAAAGGTAAAGTCAGAGCTGCAGAGCTGTTGTCCAGGGGTGAAGACACAAAGGCCCAGTGGCAGGTCTTTTCTCCCCCAAAGCCGATACCACCTGCAGGGCCCGGGCCTCTCCTGA
Del 4   GGGATAAAGGTAAAGTCAGAGCTGCAGAGCTGTTGTCCAGGGGTGAAGACACAAAGGCCCAGTGGCAGGTCTTTTCTCCCCCAAAGCCGATACCACCTGCAGGGCCCGGGCCTCTCCTGA

                                            HP                                         Meis
WT      GTGTCCCAGCACCACTCTCCAAATGATGATTAATCACGGAGTGCTAAGAGGGGGGGGGTGGTTGCACCCTGACACAGCCATAATGCTGGTCACCAGAGTCTGGATTGGCCAGCGGTGGTC
Del 4   GTGTCCCAGCACCACTCTCCA--------------------------------------------------------------------------GAGTCTGGATTGGCCAGCGGTGGTC

           Sp               HP
WT      AGTGGGTGGATGTCCGAGGAAGATGGATGACCTCGACCTCCTGTCATTAACAAGAGAAGAAACAAAACAGACAACAAAAAGAAAGGCAGCACGGTGTGAGGGAGAGTTTGTGTGGGAATA
Del 4   --------------CGAG----------------GACCTCCTGTCATTAACAAGAGAAGAAACAAAACAGACAACAAAAAGAAAGGCAGCACGGTGTGAGGGAGAGTTTGTGTGGGAATA
```

```
WT      GGGATAAAGGTAAAGTCAGAGCTGCAGAGCTGTTGTCCAGGGGTGAAGACACAAAGGCCCAGTGGCAGGTCTTTTCTCCCCCAAAGCCGATACCACCTGCAGGGCCCGGGCCTCTCCTGA
Del 5   GGGATAAAGGTAAAGTCAGAGCTGCAGAGCTGTTGTCCAGGGGTGAAGACACAAAGGCCCAGTGGCAGGTCTTTTCTCCCCCAAAGCCGATACCACCTGCAGGGCCCGGGCCTCTCCTGA

                                            HP                                         Meis
WT      GTGTCCCAGCACCACTCTCCAAATGATGATTAATCACGGAGTGCTAAGAGGGGGGGGGTGGTTGCACCCTGACACAGCCATAATGCTGGTCACCAGAGTCTGGATTGGCCAGCGGTGGTC
Del 5   GTGTCCCAGCACCACTCTCCTGCGTCAATGTAATCGGAAGAGCGTTCCC-----------------------------------------------------------------------

           Sp               HP
WT      AGTGGGTGGATGTCCGAGGAAGATGGATGACCTCGACCTCCTGTCATTAACAAGAGAAGAAACAAAACAGACAACAAAAAGAAAGGCAGCACGGTGTGAGGGAGAGTTTGTGTGGGAATA
Del 5   -------TACACGACGCTCGACCTGGATGACCTCGACCTCCTGTCATTAACAAGAGAAGAAACAAAACAGACAACAAAAAGAAAGGCAGCACGGTGTGAGGGAGAGTTTGTGTGGGAATA
```

S3 Fig. Analysis of somatic deletions.

(A) PCR amplification of either wild type (WT) embryos or F0 injected embryos (MUT), using the primers indicated in Table 1. F0 embryos were injected at the one-cell stage with CRISPR/Cas9 ribonucleic complexes targeting elements A, C, D, E, F or the krox20 gene and PCR amplifications were performed on whole embryos at stages 12s (for element A, and the krox20 gene) or at 22s (for elements C, D, E and F), corresponding to stages of in situ hybridization analysis. Complete elimination of the wild type diagnostic fragments demonstrates the high efficiency of the procedure. (B) Sequence alignments of wild type element C (WT) with five mutated alleles generated by CRISPR/Cas9 injections. Red brackets indicate the limits of the deletions and putative binding sites for transcription factors are indicated: Hox/Pbx (HP) in purple, Meis in pink, and Sp in orange.
