# Supplementary material for: Cooperation, cis-interactions, versatility and evolutionary plasticity of multiple cis-acting elements underlie krox20 hindbrain regulation
Source: PLoS Genet. 2018 Aug 6;14(8):e1007581. doi: 10.1371/journal.pgen.1007581 (PMC6095606; doi:10.1371/journal.pgen.1007581)
Supplement: S4 Fig — Embryos from three lines carrying homozygous deletions of a cis-acting element (∆) or F0 embryos carrying somatic mutations (*) in the same elements were analysed for the expression of krox20 by in situ hybridization at the indicated stages. WT, wild type control. Positions of r3 and r5 are shown. (PDF) [file pgen.1007581.s004.pdf]

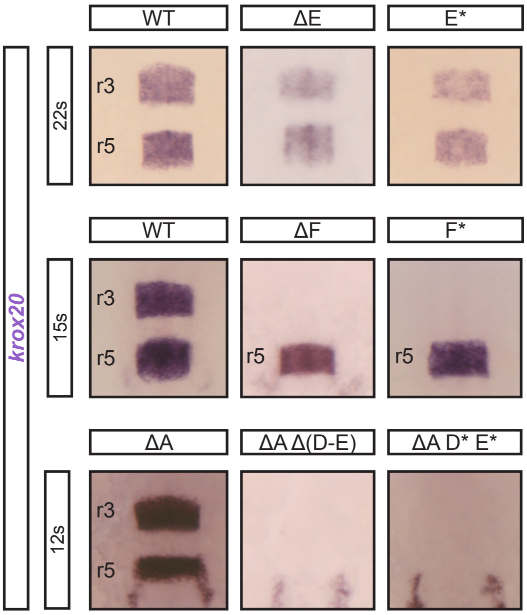

S4 Fig. Very similar phenotypes obtained following germ-line or somatic deletion.
Embryos from three lines carrying homozygous deletions of a cis-acting element (Δ) or F0 embryos carrying mutations (*) in the same elements were analysed for the expression of krox20 by in situ hybridization at the indicated stages. WT, wild type control. Positions of r3 and r5 are shown.
