# Supplementary material for: Cooperation, cis-interactions, versatility and evolutionary plasticity of multiple cis-acting elements underlie krox20 hindbrain regulation
Source: PLoS Genet. 2018 Aug 6;14(8):e1007581. doi: 10.1371/journal.pgen.1007581 (PMC6095606; doi:10.1371/journal.pgen.1007581)
Supplement: S5 Fig — Oligonucleotides corresponding to sequences present in enhancers A, D and E and covering Krox20 binding sites (see the Materials and Methods section for the sequences of the oligonucleotides and S2 Fig for the positions of the Krox20 binding sites) were subjected to polyacrylamide gel retardation assays. Biotin-double-stranded oligonucleotides were exposed to bacterial (Pet) extracts containing the Krox20 protein or not (-), in the presence or absence (-) of an unlabelled oligonucleotide competitor. The competitor oligonucleotides carried either a bona fide Krox20 binding site (WT) or a mutated version (Mut) that does not allow Krox20 binding. The arrows indicate the migration positions on the gel of the free oligonucleotides (free probe) and of retarded bands corresponding to specific complexes with the Krox20 protein. (PDF) [file pgen.1007581.s005.pdf]

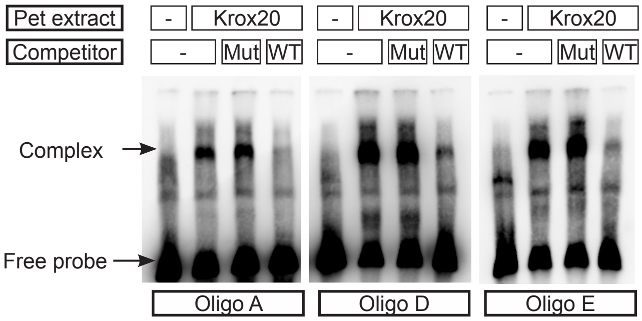

S5 Fig. Presence of Krox20 binding sites within enhancers A, D and E.
Oligonucleotides corresponding to sequences present in enhancers A, D and E and covering Krox20 binding sites (see the Materials and Methods section for the sequences of the oligonucleotides and Supplementary Fig. S1 for the positions of the Krox20 binding sites) were subjected to polyacrylamide gel retardation assays. Biotin-double-stranded oligonucleotides were exposed to bacterial (Pet) extracts containing the Krox20 protein or not (-), in the presence or absence (-) of an unlabelled oligonucleotide competitor. The competitor oligonucleotides carried either a bona fide Krox20 binding site (WT) or a mutated version (Mut) that does not allow Krox20 binding. The arrows indicate the migration positions on the gel of the free oligonucleotides (free probe) and of retarded bands corresponding to specific complexes with the Krox20 protein.
