# Supplementary material for: Cooperation, cis-interactions, versatility and evolutionary plasticity of multiple cis-acting elements underlie krox20 hindbrain regulation
Source: PLoS Genet. 2018 Aug 6;14(8):e1007581. doi: 10.1371/journal.pgen.1007581 (PMC6095606; doi:10.1371/journal.pgen.1007581)
Supplement: S6 Fig — Transgenic 12s embryos carrying the GFP reporter driven by the A enhancer were either uninjected (Control) or injected with Cas9 and guide RNAs targeting the coding sequence of Krox20’s zinc fingers (krox20*) and analysed by single in situ hybridization with a krox20 probe (purple labelling) or double in situ hybridization with krox20 (purple labelling) and GFP (orange labelling) probes. (PDF) [file pgen.1007581.s006.pdf]

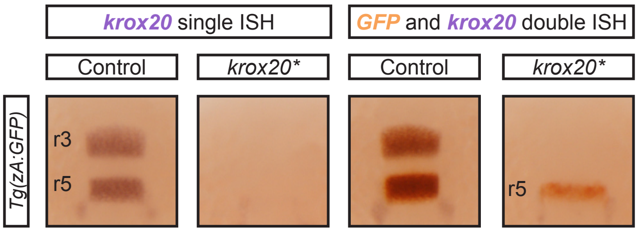

S6 Fig. Loss of Krox20 activity in embryos injected with guide RNAs targeting sequences encoding Krox20's zinc fingers.

Transgenic 12s embryos carrying the GFP reporter driven by the A enhancer were either uninjected (Control) or injected with Cas9 and guide RNAs targeting the coding sequence of Krox20's zinc fingers (krox20*) and analysed by single in situ hybridization with a krox20 probe (purple labelling) or double in situ hybridization with krox20 (purple labelling) and GFP (orange labelling) probes.
