# Supplementary material for: Cooperation, cis-interactions, versatility and evolutionary plasticity of multiple cis-acting elements underlie krox20 hindbrain regulation
Source: PLoS Genet. 2018 Aug 6;14(8):e1007581. doi: 10.1371/journal.pgen.1007581 (PMC6095606; doi:10.1371/journal.pgen.1007581)
Supplement: S1 Table — (PDF) [file pgen.1007581.s007.pdf]

Sequence	ZED Cloning primers	CRISPR/Cas9<br>germline deletion guides	CRISPR/Cas9<br>somatic deletion guides	Genotyping primers
zA	Forward: 5'-AGAGCCTATGTTGGAAACAACAC-3'<br>Reverse: 5'-TAGCGTAGTAGGCCCAGTAAAG-3'	5'-GGAGAGACAATTTTTTTATG-3'<br>5'-GGATACATCGGTGCCGCTTTAG-3'	5'-TCTTTTAGGGCGCTGTGTGT-3'<br>5'-CACTGGAATCTGCGTGTAGG-3'<br>5'-TGTGCTGACAGAAGAAGAGC-3'<br>5'-AAATGCAGAGTAAAACAATT-3'	Forward: 5'-GTGTGGGAAGCAAATTATAGA-3'<br>Reverse: 5'-GGGTTAGGTGTACACATTCATA-3'
zB	Forward: 5'-GGTAAAATCTCCCACCATCGA-3'<br>Reverse: 5'-CACCGCCGAAAAACAATAGCG-3'	5'-GATAAAGGGGGAGTTTAC-3'<br>5'-GGACCACTTCAAACATTGTT-3'	NA	Forward: 5'-ACCATCGACCCACACCTAAA-3'<br>Reverse: 5'-CCGACCAACCAACCCACAGA-3'
zC	Forward: 5'-GGGATAAAGGTAAAGTCAGAGCT-3'<br>Reverse: 5'-TGGGTCATTAGGGGGATTGTG-3'	5'-GGCCTTTGTGTCTTCACCCC-3'<br>5'-GGAATAAAAGAATAGGTAGA-3'	5'-CCGGGCCCTGCAGGTGGTAT-3'<br>5'-CTCCGTGATTAATCATCATT-3'<br>5'-ACCAGCATTATGGCTGTGTC-3'<br>5'-GGCCAGCGGTGGTCAGTGGG-3'	Forward: 5'-ACTCCAACCGCTGGGTTAAA-3'<br>Reverse: 5'-CACGCTCTGGTTTCCTCCAA-3'
zD	Forward: 5'-CTTTCCCAAAACCAAAGTGAGCCT-3'<br>Reverse: 5'-GCAAGCTGTTTTAAGTCAGGTTAAC-3'	5'-ACCCTCATCTAAATTTTTGT-3'<br>5'-TGTTGTTGCGGAGTCTCCCA-3'<br>5'-TGTTCTTTTAACCGAGTAGG-3'<br>5'-TTTTCTTAGTGATTTTGTGT-3'	5'-ACCCTCATCTAAATTTTTGT-3'<br>5'-TGTTGTTGCGGAGTCTCCCA-3'<br>5'-TGTTCTTTTAACCGAGTAGG-3'<br>5'-TTTTCTTAGTGATTTTGTGT-3'	Forward: 5'-CATGGAATCTATTTTCTTTCCC-3'<br>Reverse: 5'-GCTAGCGAAACTTATACAATG-3'
zE	Forward: 5'-CACGTTTTTACGCAGCGG-3'<br>Reverse: 5'-TTGAGAGTGATCTGGCTC-3'	5'-TAGCTGCAAGTGTGGTCTTT-3'<br>5'-AACCCAATGCTTATAATTCT-3'	5'-CCTTCTTTCATTTGTGGGGG-3'<br>5'-GCAAACATTTTTGCATCTGT-3'<br>5'-ATTCAGTGGCTGCCATGTGT-3'	Forward: 5'-CGCAACCCATCTCTGGGAAAT-3'<br>Reverse: 5'-TGGCTCCATAGTTAAGCTGCA-3'
zF	Forward: 5'-GATTCATCACTCCAACCTTTTC-3'<br>Reverse: 5'-ACCACAGTACGACGGTAGAG-3'	5'-TTGCATATCACTAAAGACAA-3'<br>5'-GATATGACAATGACAATATG-3'	5'-CTTGATCCTCAACAATAACA-3'<br>5'-TGTGGAGAACACGACAGAGA-3'<br>5'-AGCTCTCTCTCCCCTCCAGG-3'	Forward: 5'-TGCTAGCTGGGCTATTTCACAC-3'<br>Reverse: 5'-ACCAACCAGTGATACAGTAAGTG-3'
(D-E)	NA	5'-TAGCTGCAAGTGTGGTCTTT-3'<br>5'-TTCCCAAGTGCTTTTGTAAA-3'	NA	Forward: 5'-CGCAACCCATCTCTGGGAAAT-3'<br>Reverse: 5'-GCTAGCGAAACTTATACAATG-3'
(A-E)	NA	5'-ACGGACAACAGGAAGCTATG-3'<br>5'-CATTAGGGTTTTCTATCCAG-3'	NA	Forward: 5'-CTGTCAATCTATGGACAGAGAAC-3'<br>Reverse: 5'-GCTGTCCAAACACTGTGTGC-3'
Egr2b	NA	NA	5'-TTTGCGGGAGAAAGTTCGCG-3'<br>5'-TGCGAATGTGGGTCGTCAGG-3'<br>5'-GGTGTGGATGCGGATGTGTC-3'	Forward: 5'-TGGTTTGAACTGGACGAGCAG-3'<br>Reverse: 5'-CAAGGAAATACCCCAACAGACC-3'
kA	Forward: 5'-TTGTGGGATAATGCCTCCTGTA-3'<br>Reverse: 5'-GTAGGCCTAATTCACACCAATTG-3'	NA	NA	NA
sA	Forward: 5'-CTTATTGCTTCATTTGAGGGAAGG-3'<br>Reverse: 5'-GACTCATTGAAAACCCAGCATAAAG-3'	NA	NA	NA
xA	Forward: 5'-GAGGCAAAGTCTTCTTGCATTGCTC-3'<br>Reverse: 5'-GGAGTTTAGCAACAATGGCTGGAG-3'	NA	NA	NA
cA	Forward: 5'-CAGCTAAGGGAAGTGCCAAATC-3'<br>Reverse: 5'-CTAAAATCCTGGTGTGCTGG-3'	NA	NA	NA
mA	Forward: 5'-CCACCTGACAGGTTGACTTC-3'<br>Reverse: 5'-GTTGGCTTCAACGAGGTTTTGC-3'	NA	NA	NA

S1 Table. Sequences of the 3' end of the cloning primers used for constructing the ZED constructs, the 5' end of CRISPR/Cas9 RNA guides used to create the germ-line and somatic deletions and the PCR primers used to detect the deletions.
